# Supplementary material for: Multifunctional Chitosan–Covalent Bonded Multi‐Walled Carbon Nanotubes Composite Binder for Enhanced Electrochemical Performances of Lithium–Sulfur Batteries
Source: Macromol Rapid Commun. 2025 Apr 10;46(11):2500155. doi: 10.1002/marc.202500155 (PMC12143368; doi:10.1002/marc.202500155)
Supplement: Supplementary file 1 — Supporting Information [file MARC-46-2500155-s001.docx]

**Multifunctional Chitosan–covalent Bonded Multi-walled Carbon Nanotubes Composite Binder for Enhanced Electrochemical Performances of Lithium-sulfur Batteries**

*Qiuying Gou, Liqiang Lu, Shengxuan Lin, Wei Zhang,* *Yael Rodriguez Ayllon, Zhe Zhou, Liping Zhu,* Yan Lu**

Liqiang Lu, Shengxuan Lin, Wei Zhang, Yael Rodriguez Ayllon, Yan Lu

Institute of Electrochemical Energy Storage

Helmholtz-Zentrum Berlin für Materialien und Energie

Hahn-Meitner-Platz 1, 14109 Berlin, Germany

[yan.lu@helmholtz-berlin.de](mailto:yan.lu@helmholtz-berlin.de)

Qiuying Gou, Zhe Zhou, Liping Zhu

State Key Laboratory of Advanced Fiber Materials, College of Materials Science and Engineering, Donghua University, 2999 North Renmin Rd, Songjiang, Shanghai 201620, China

[zhulp@dhu.edu.cn](mailto:zhulp@dhu.edu.cn)

Y. Lu

Institute for Technical and Environmental Chemistry, Friedrich-Schiller-Universität Jena, Philosophenweg 7b, 07743 Jena, Germany
Y. Lu

Helmholtz Institute for Polymers in Energy Applications (HIPOLE Jena), Lessingstraße 12-14, 07743 Jena, Germany

Keywords: Li-S battery, composite binder, water-based, chitosan, MWCNTs

Lithium-sulfur batteries (LSBs) are considered as one of the most promising next-generation energy-storage devices because of their high energy density. However, the long-term use of LSBs is mainly limited by polysulfide shuttling and cathode structural degradation caused by volume changes during charging and discharging. To address these issues, a multifunctional, high-performance aqueous binder is developed by modifying a natural polysaccharide with multi-walled carbon nanotubes (MWCNTs). Specifically, the catechol-conjugated chitosan (CCS) acts as the binder, showing strong polysulfide adsorption, while the MWCNTs covalently bonded to CCS enhance the mechanical toughness and electronic conductivity. The resulting CCS-MWCNTs composite binder exhibits a tensile strength of 40 MPa and a strain at break of 300%, which are higher than those of CCS. As a binder for sulfur cathodes, the CCS-MWCNTs binder demonstrates superior cyclic stability and rate capability. At a sulfur loading of 2.0 mg cm⁻², it delivers an initial capacity of 1016 mAh g⁻¹ at 0.2 C and retains 690 mAh g⁻¹ after 100 cycles, significantly outperforming commercial PVDF, CMC/SBR, and CCS binders. This study demonstrates the potential applications of polysaccharide binders in metal-sulfur batteries by innovatively incorporating carbon nanotubes into the biopolymer binder, providing a promising alternative for environmentally friendly energy storage.

# 1. Introduction

As society transitions away from fossil fuels, the demand for advanced battery technologies with high energy storage capacity and reduced reliance on critical minerals becomes increasingly urgent.^[1-4]^ Lithium-sulfur batteries (LSBs) have attracted significant research interest due to their high theoretical specific energy density (2600 Wh kg^-1^) compared to the state-of-the-art commercial lithium-ion batteries (e.g. ~250 Wh kg^-1^),^[5, 6]^ and the advantage of natural abundance of elemental sulfur. However, the performance of LSBs deteriorates rapidly due to several factors, including the insulating nature of sulfur and lithium sulfide, the notorious “shuttle effect” caused by dissolved polysulfides, and significant volume expansion of up to 80% that induces high mechanical stress and rich microcracks during lithiation-delithiation cycles.^[7, 8]^ These challenges collectively hinder the commercial viability of LSBs.

In LSB cathodes, polymeric binders can play a crucial role in stabilizing sulfur and conductive carbon within the liquid electrolyte, helping to mitigate some of the challenges and improve battery performance.^[9-14]^ However, conventional binders such as polyvinylidene fluoride (PVDF), are primarily designed for lithium-ion batteries and suffer from inadequate electronic and ionic conductivity, as well as poor polysulfide adsorption ability,^[15]^ inadequately to trap polysulfides from dissolution and then migration into bulk electrolyte. Furthermore, the slurry preparation of PVDF binders requires N-methyl-2-pyrrolidone (NMP), which is a highly toxic and costly solvent.^[16-18]^

In contrast, aqueous biopolymer binder offers more functions and are environmentally friendly alternatives. Commercial aqueous binders like sodium carboxymethyl cellulose/styrene-butadiene rubber (CMC/SBR) are commonly used,^[19, 20]^ along with new options such as fenugreek gum,^[21]^ alginate,^[22]^ pectin,^[23]^ , guar gum,^[24]^ and dextrin.^[25]^ These materials not only contain functional groups such as hydroxyl, carboxyl, and ether groups, which are highly effective in adsorbing polysulfides^[26, 27]^, but also possess advantages like environmental sustainability, abundant availability, and ease of procurement^[28]^. Such features make biomacromolecules highly promising for lithium-sulfur battery applications.

Chitosan (CS), a natural polysaccharide composed of β-(1,4)-linked 2-deoxy-2-amino-D-glucose units,^[29]^ has gained considerable attention due to its abundance and unique chemical structure. It contains numerous amino and hydroxyl functional groups, which can effectively immobilize polysulfides through hydrogen bonding and chemical adsorption, thereby mitigating the shuttle effect.^[30]^ Additionally, the flexible ether bonds in chitosan enable efficient lithium-ion transport,^[31]^ improving electrochemical performances. These favorable properties have led to extensive research into the application of chitosan for LSBs.^[32-36]^ However, the overall performance of the chitosan-based binders still requires improvement. For example, Ramos-Sanchez et al.^[34]^ introduced various ratios of sulfonic acid groups into the chitosan molecular chains and prepared tensile samples for testing. They found that none of the samples experienced a strain greater than 1.2%, indicating insufficient toughness. In addition, the synthesis methods reported in these binders are often complex,^[32]^ difficult to be scaled up for industrial applications, or reliant on toxic solvents.

Conductivity is another critical property for binders.^[37]^ Carbon nanotubes have garnered considerable attention in LSBs due to their superior electrical conductivity and mechanical strength, which can significantly enhance the overall performance of electrode materials when used as sulfur hosts or conductive additives.^[38-40]^ However, these materials are often insoluble in pure water or incompatible with the specific requirements of lithium-sulfur battery binders.^[41-45]^ Therefore, there is a strong need to explore and develop aqueous biopolymer binders specifically tailored for LSB applications, offering high mechanical strength, good electronic conductivity, and enhanced polysulfide adsorption capabilities.
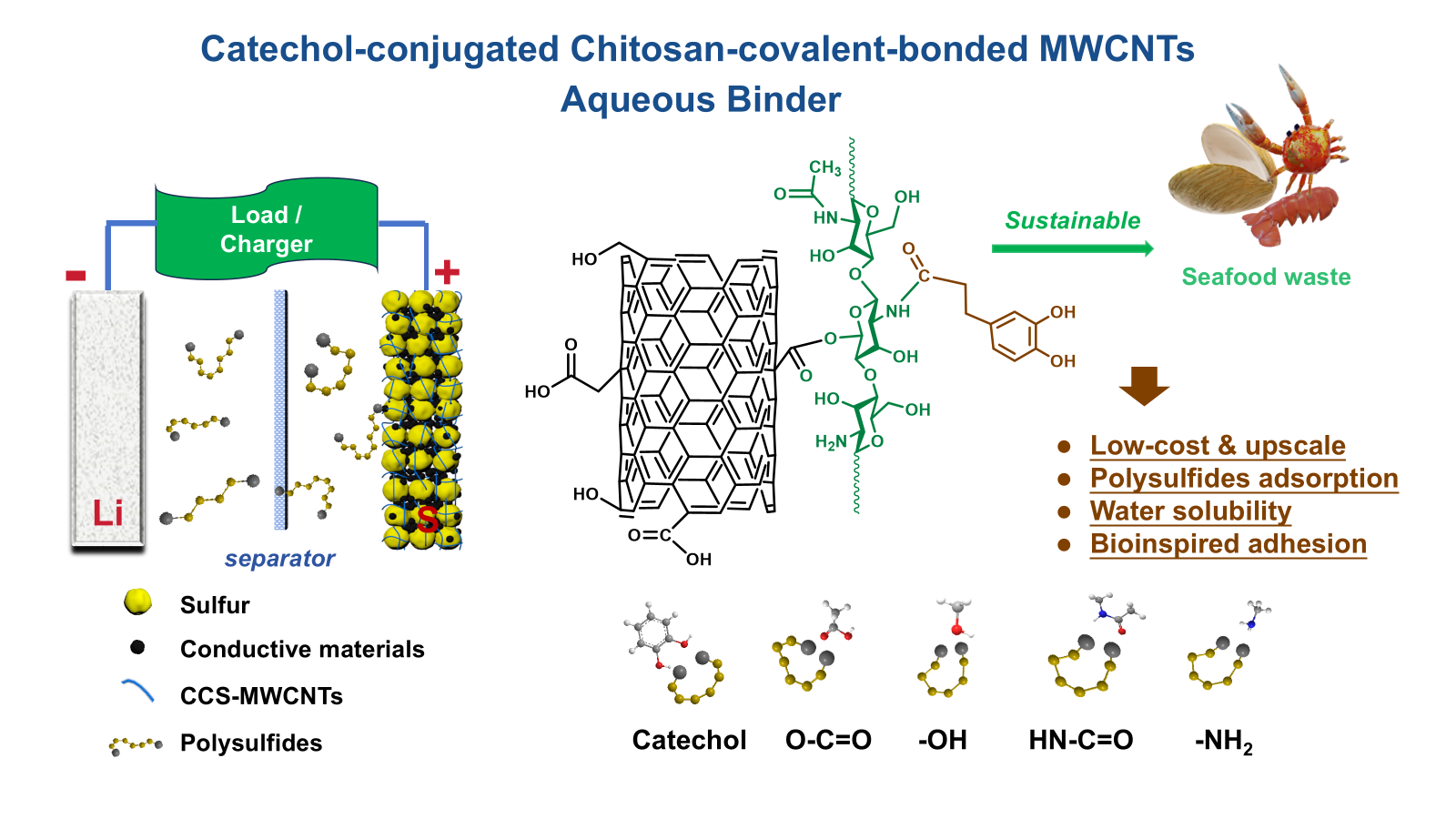


**Figure 1**. Schematic illustration of the designed molecular structure and working mechanism of the aqueous binder of catechol-conjugated chitosan-covalent-bonded MWCNTs (CCS-MWCNTs) with multiple functions for lithium-sulfur batteries.

In this study, a novel functional binder was designed as schematically shown in **Figure 1** and developed with modified chitosan and carbon nanotubes. 3,4-dihydroxyhydrocinnamic acid (HCA), as a natural phenolic compound found in various plants, contains catechol groups that exhibit strong adhesion properties. Inspired by the nature adhesion properties of HCA, this study first grafted HCA onto the chitosan backbone to improve the adhesion property and solubility of chitosan. Meanwhile, as a proof of concept, multi-walled carbon nanotubes (MWCNTs) were chemically modified to introduce carboxyl and hydroxyl functional groups, enhancing their chemical interaction with the modified chitosan, improving their dispersion within the chitosan matrix, and strengthening the toughness and conductivity of the composite binder. Afterwards, by mixing the modified chitosan aqueous solution and surface-functionalized carbon nanotube dispersion under heat, the multi-functional composite binder with enhanced mechanical and electrical properties was obtained. This new biopolymer composite was investigated as a binder for sulfur cathode, and demonstrated excellent electrochemical performance, providing an effective solution for producing high-quality sulfur cathodes and thus improving the performance of lithium-sulfur batteries.

# 2. Results and Discussion

Frist of all, to improve the water solubility of chitosan and to enhance its capability of polysulfide adsorption, hydrogenated caffeic acid (HCA) was grafted onto the chitosan molecular chains through an amination reaction. **Figure 2a** illustrates the amide bonds formed between the amino groups of chitosan and the carboxyl groups of HCA. The nuclear magnetic resonance (NMR) spectrum in the Supplementary Information (**Figure S1**) shows that a grafting ratio of 15% was obtained in this work. Meanwhile, MWCNTs were surface functionalized with hydroxyl groups through an oxidation method using concentrated nitric acid, enabling MWCNTs stabilized in water and to form covalent bonds with HCA-modified chitosan. The transmission electron microscopy (TEM) image in **Figure 2b** demonstrates that the original MWCNTs exhibit a dense and aggregated structure with limited separation between individual carbon nanotubes. In contrast, the modified MWCNTs form a more dispersed and looser network, where individual nanotubes are clearly visible, and the degree of aggregation is significantly reduced. This enhanced dispersion facilitates a uniform mixing of the carbon nanotubes with the modified chitosan, promoting stable interactions that substantially improve the mechanical and electrical properties of the formed composite binder. Such improvements are crucial for accommodating the volume changes that occur during charge and discharge cycles while maintaining conductivity. Fourier transform infrared spectroscopy (FT-IR) characterization (**Figure 2c**) provides key information on pure chitosan (CS), catechol-conjugated chitosan (CCS), and CCS-MWCNTs. The absorption peaks at 1650 cm^-1^ and 1591 cm^-1^ in chitosan correspond to the stretching vibration of the N-acetyl group (amide I) and the bending vibration of NH (amide II) of chitosan, respectively.^[46]^ After HCA grafting, these peaks shift to lower wavenumbers, potentially due to the formation of denser hydrogen bonding and the introduction of aromatic ring structures, which create steric hindrance for restriction of molecular rotation. Additionally, the appearance of a new C=O peak at 1740 cm^-1^ and the weakened peak of amino groups at 1320 cm^-1^ suggest the formation of covalent bonds between the carboxyl group of HCA and the amino group of chitosan,^[47, 48]^ further confirming the successful grafting modification. In the CCS-MWCNTs composite, the broadened C=O peak at 1740 cm^-1^ and the enhancement of the C-O peak at 1250 cm^-1^ indicate the formation of ester bonds between the modified chitosan and the functionalized carbon nanotubes.^[49]^ Moreover, a comparison of the XPS O 1s spectra of CCS and CCS-MWCNTs (**Figure S2**) reveals that CCS-MWCNTs exhibits a new peak at 533.7 eV, corresponding to ether-type oxygens in ester groups.^[50]^ This further confirms the hybridization of the carbon nanotubes and CCS.

Thermogravimetric analysis (TGA) was employed to evaluate the thermal stability of the samples. As shown in **Figure 2d**, two distinct degradation stages have been observed for all three materials. The mass loss below 150 °C in CCS and CCS-MWCNTs is primarily attributed to the physisorbed water and the variable moisture content left in the samples, while the second degradation stage occurs between 150 °C and 350 °C, involved with the thermal decomposition of the CCS backbone and the breakdown of the carbon nanotubes in the composite. The grafted functional groups affect the original crystallinity of CS, resulting in a lower decomposition temperature for CCS compared to pure CS. However, the mass loss rate of CCS and CCS-MWCNTs between 250-350 °C is slower, indicating that the amide bond and ester bond formed during the grafting reaction and the introduction of carbon nanotubes exhibit different thermal degradation behavior. At 800 °C, the residual mass of CCS-MWCNTs is 41.7%, higher than that of CCS (36.5%) and CS (33.1%). This difference is attributed to the different carbon contents in the grafted catechol groups and MWCNTs.

Considering that the binder system in Li-S batteries needs to accommodate around 80% volume expansion of sulfur electrodes, mechanical performance is therefore a critical factor for binder evaluation. In this context, films of CCS and CCS-MWCNTs were prepared, and their mechanical properties were assessed. The optical images in **Figure 2e** demonstrate the excellent film-forming ability and flexibility of the CCS-MWCNTs composite material. Good integrity was maintained under folding and bending. Additionally, tensile strain tests have been conducted to evaluate its mechanical properties and compared with commercial PVDF and CMC/SBR binders. Figure S3 illustrates the stress–strain relationship, which enables the determination of ultimate tensile strength (UTS) and Young’s modulus. CCS showed poor ultimate tensile strength. By the introduction of 3 wt% MWCNTs, CCS is reinforced and exhibited higher fracture strength and higher toughness than CCS. However, when the MWCNTs content reaches 6%, the toughness and fracture strength become similar with those of CCS. This may be due to the exacerbation of nanotube aggregation, leading to stress concentration during loading and premature fracture. Therefore, we selected the sample with 3 wt% MWCNTs (CCS@MWCNTs-3) for further comparison with commercial PVDF and CMC/SBR binders in tensile strain tests to evaluate its mechanical properties and electrochemical performances. As shown in **Figure 2f**, PVDF displays the highest toughness (tensile strain up to 65%), but its fracture stress was only 3.17 MPa, indicating limited load-bearing capacity. In contrast, CCS exhibits the lowest toughness (3%) but with a fracture strength of 29.98 MPa, suggesting potential applications under high-load conditions. CMC/SBR has shown slightly higher fracture strength than CCS, and the addition of SBR increases its tensile strain, improving the flexibility. Notably, the CCS-MWCNTs binder, through covalent bonding of CCS with only 3 wt.% MWCNTs, demonstrates the highest elastic modulus and maximum tensile strength (37.96 MPa), indicating that the introduction of carbon nanotubes significantly enhances the mechanical properties of CCS, while showing good interfacial compatibility. Therefore, this modification endows CCS-MWCNTs binder with superior tolerance to sulfur volume changes compared to PVDF and CMC/SBR binders, potentially contributing to improved cycling stability and minimized structural damage upon discharge-charge cycling in Li-S batteries.


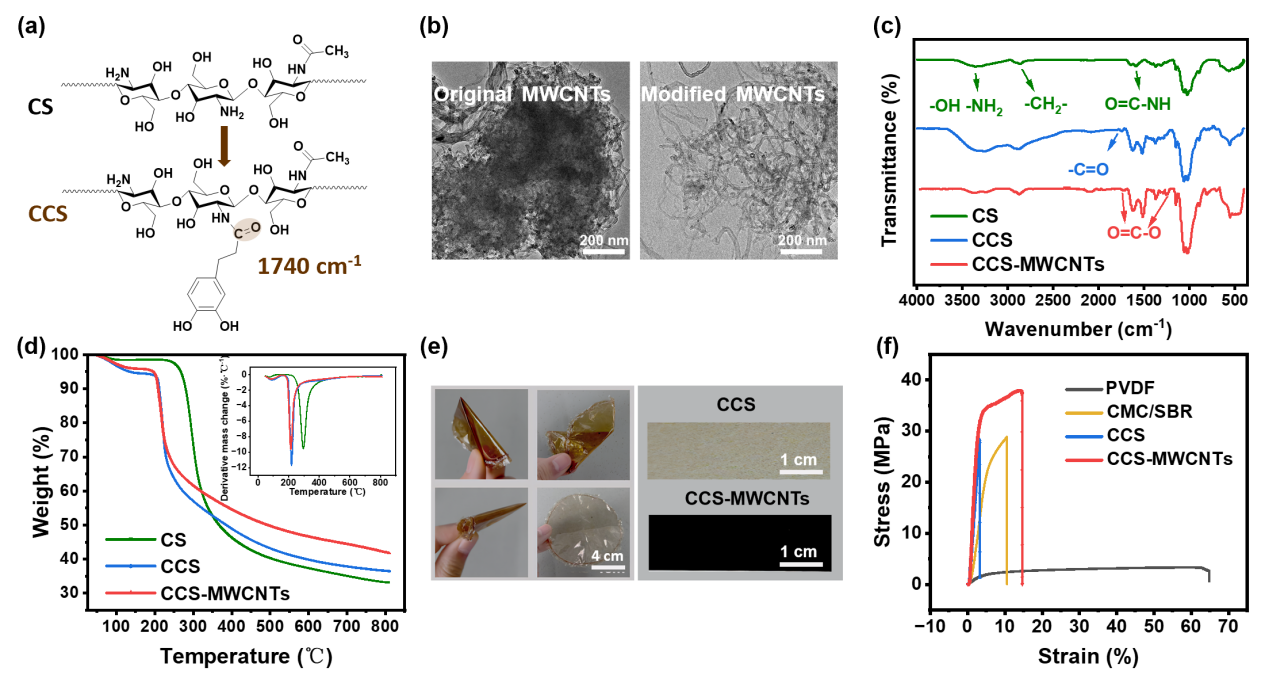


**Figure 2.** Characterization and properties of the biopolymer composite binders: (a) chemical structures of CS and CCS binder, (b) TEM images of the MWCNTs before and after oxidation treatment, (c) FTIR spectra of CS, CCS, and CCS-MWCNTs binders, (d) TGA and DTG curves of CS, CCS, and CCS-MWCNTs, (e) photos showing the bending flexibility of the CCS film and photos of CCS and CCS-MWCNTs films, and (f) mechanical properties of different binder films.

The modification of chitosan with catechol (HCA grafting) increases its solubility in water and improves its thermal stability and mechanical properties. As a result, catechol-conjugated chitosan and the CCS-MWCNTs composite materials show great potential for producing high-quality electrodes. The cathodes were prepared by the method of doctor-blade casting of slurry. The slurry formation was conducted by mixing Ketjen black-sulfur (KB/S, 80 wt% of sulfur content) composite, Super P conductive and different binders with a mass ratio of 7:2:1. For commercial PVDF binder, the slurry was prepared with NMP as solvent, while water was used for all the other three binder (CMC/SBR, CCS, CCS-MWCNTs). It should be also mentioned that Ketjen black carbon, which is notable for its challenging in thick-sulfur-cathode preparation due to its high specific surface area and highly porous structure, was selected as the sulfur host material to examine the binding performance of various binders. **Figure 3** presents the scanning electron microscope (SEM) images of the sulfur cathodes prepared with four different binders with two different sulfur loadings: 1 mg cm^-2^ shown in **Figure 3 a-d** and 2 mg cm^-2^ shown in **Figure 3 e-h.** The SEM images reveal distinct morphological features and structural integrity associated with each binder type and sulfur loading. For cathodes with 1 mg cm^-2^ of sulfur loading, PVDF- and CMC/SBR-based cathodes show plenty of microcracks. The CCS- and CCS-MWCNTs-based cathodes present almost crack-free and homogeneous coatings, demonstrating its excellence of structural integrity and great potential of binder applications. When increasing the sulfur loading to 2 mg cm^-2^, the PVDF cathode exhibits large-size and eye-visible macroscopic cracks (approximately 100 micrometers), indicating that PVDF binder performance is insufficient under higher sulfur loading conditions. In contrast, aqueous binder-based cathodes demonstrate smaller microcracks. For the CMC/SBR electrodes, although the number of micro-cracks decreases with the increased sulfur loading, the crack width becomes significantly larger. In contrast, electrodes prepared with CCS and CCS-MWCNTs exhibit fewer and smaller-depth micro-cracks, particularly the CCS-MWCNT electrode. By calculating the ratio of overall micro-crack area on the entire electrode area with sulfur loading of 1 mg cm^-2^, the defect rates of the electrodes for the four binders (PVDF, CMC/SBR, CCS, and CCS-MWCNTs) have been obtained, which are 4.43%, 4.85%, 0.17%, and 0.04%, respectively (**Figure S3**), reflecting different quality of binders. The above results indicate that the CMC/SBR binder results in the highest number of micro-cracks, likely due to poor compatibility between CMC and SBR. Although the PVDF electrodes exhibit fewer micro-cracks than that of the CMC/SBR electrodes, the depth and sizes of these cracks are significantly larger, indicating more severe cracking. In comparison, CCS and CCS-MWCNT exhibit the lowest defect rate, demonstrating the excellent electrode quality and superior binder performance.

The optical images in **Figure 3i** show the different quality of cathodes, further demonstrating that CCS-MWCNT binder is more beneficial not only for crack-less structure than PVDF, but also for being potentially applicable for large-area electrode preparation under high sulfur loading conditions. This may be attributed to the superior mechanical properties of the CCS-MWCNT binder and its strong interfacial adhesion with the electrode materials, promoting a more uniform distribution of sulfur particles. Chitosan is a natural hydrophobic linear macromolecule, and the presence of catechol allows these molecular chains to dissolve in water. At the same time, the main chain of chitosan remains hydrophobic, enabling it to adsorb onto similar sulfur-carbon particles via hydrophobic interactions, thereby preventing the agglomeration of active materials and forming a more homogeneous active slurry. The addition of MWCNTs further enhances the mechanical toughness of the binder, allowing it to withstand volume shrinkage during electrode drying. Such excellent uniformity is crucial for optimizing electrochemical performance, as it enhances both the overall conductivity of the electrodes and their mechanical stability, thereby would be helpful for improving the rate performance and extending cycle life of the battery.


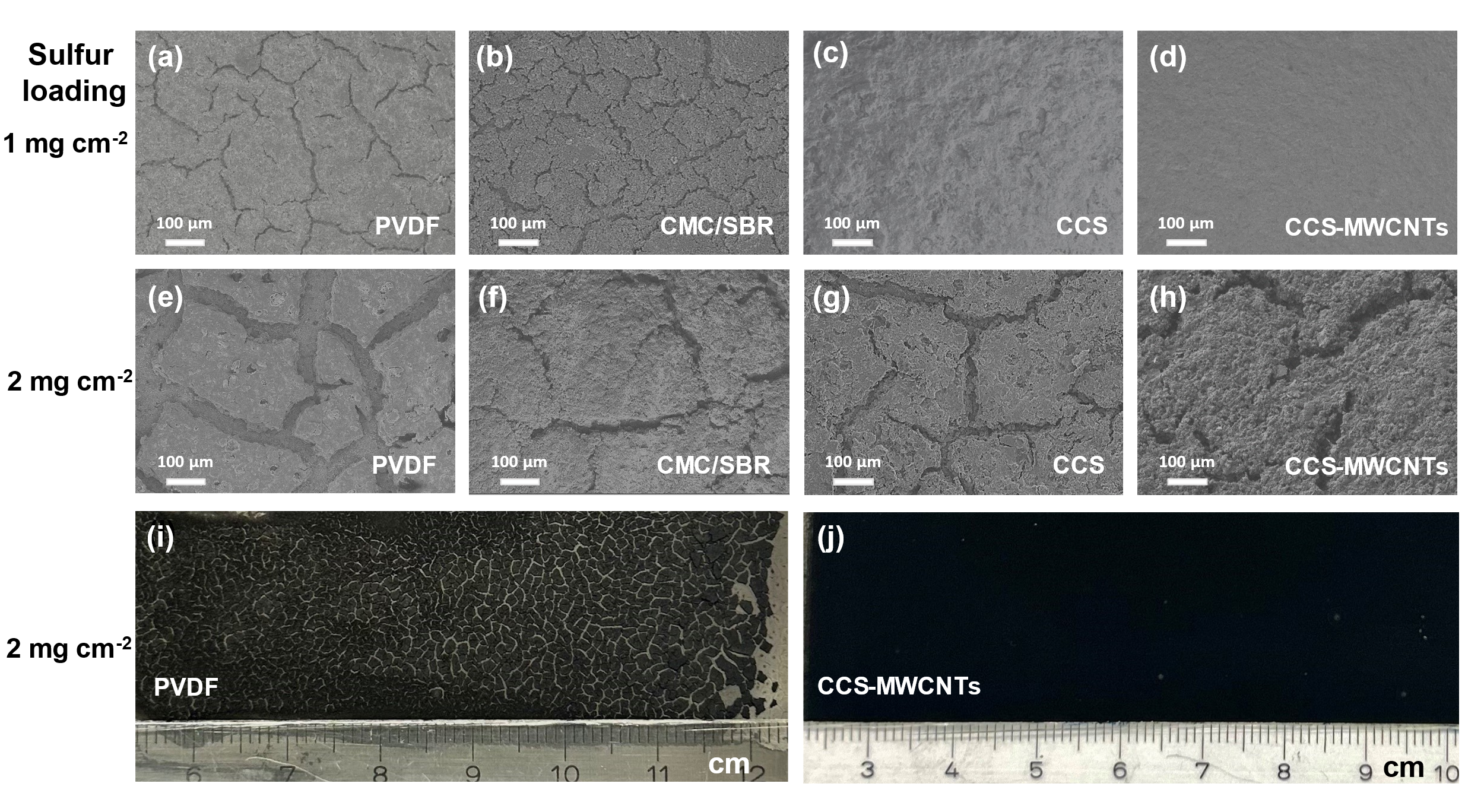


**Figure 3**. SEM images of the cathodes prepared with different binders with areal sulfur loadings: (a-d) 1 mg cm^-2^, (e-h) 2 mg cm^-2^. Photos of (i) PVDF and (j) CCS-MWCNT electrodes with sulfur loading of 2 mg cm^-2^.

Chemical adsorption has been proven to be an effective strategy towards suppressing shuttling of polysulfides.^[51, 52]^ **Figure 4(a-d)** presents the XPS spectra of CCS. **Figure 4a** shows the C 1s spectrum, which can be deconvoluted into four peaks: the peak at 285.6 eV corresponds to C–C bonds, 284.5 eV indicates the presence of C=C bonds,^[53]^ confirming the successful grafting of hydrogenated caffeic acid onto the chitosan molecular chains. The peaks at 286.5 eV and 288 eV are attributed to C–O and C=O bonds, respectively.^[54]^ **Figure 4b** shows the N 1s XPS spectra of CCS, with three different peaks centered at 399.5, 400.7, and 401.8 eV, corresponding to C-NH_2_, O=C-N, and C-NH_3_^+^, respectively.^[55]^ In **Figure 4c**, the measured O 1s binding energies were 531.1 eV for C=O in N-acetylated-glucosamine units and 532.9 eV for C–O bonds corresponding to ether and alcohol oxygen.^[56]^ These functional groups contribute to strong chemical adsorption of polysulfides. After immersing CCS in a 20 mM Li_2_S_6_ solution followed with drying, the comparison of the XPS spectra before and after adsorption, as shown in **Figure 4d**, indicates the emergence of peaks corresponding to sulfur (S) and lithium (Li) in the post-adsorption spectrum (CCS-LiPS).


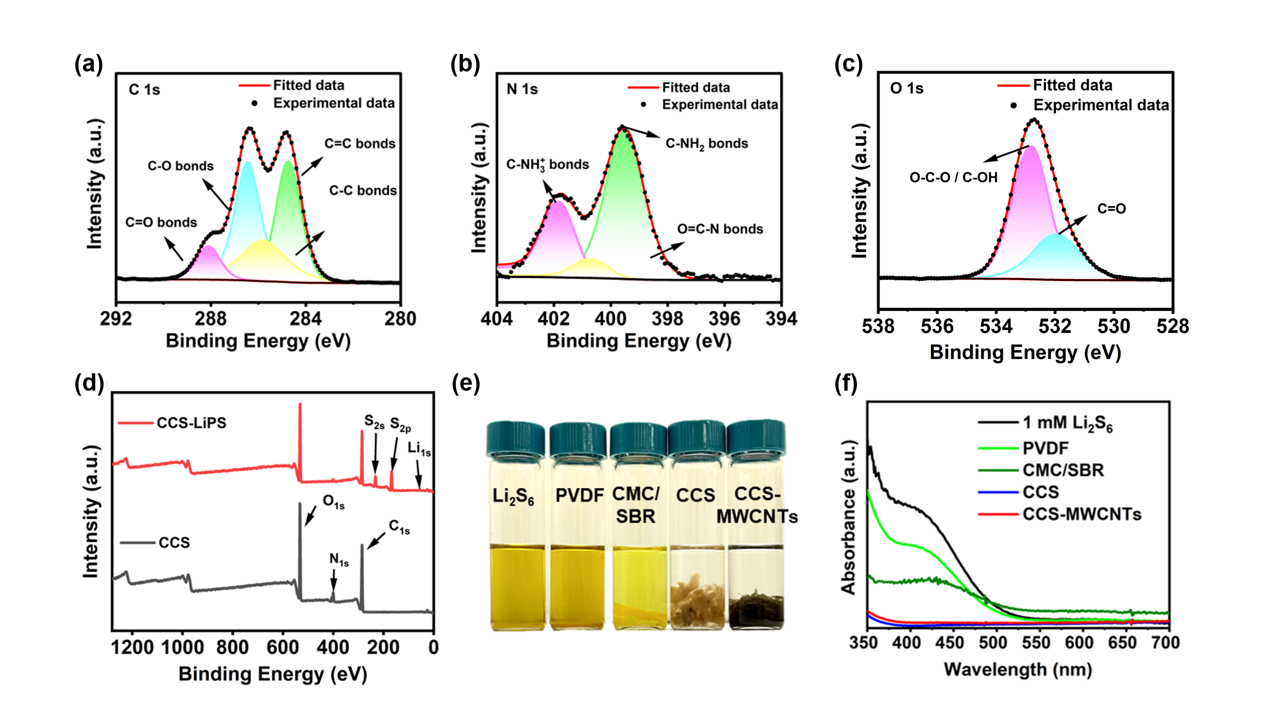


**Figure 4**. XPS spectra of CCS binders: (a) C 1s, (b) O 1s, (c) N 1s, and (d) full spectra before and after adsorption. Polysulfide adsorption: (e) Photos of 10 mg of the four types of binders in 1 mM of Li_2_S_6_ solutions. (f) UV–Vis absorption spectrum of Li_2_S_6_ in DOL/DME electrolyte solution with four types of binders after 4 hours.

The detailed spectra of CCS after polysulfide adsorption are shown in the Supplementary Information. **Figure S4** compares the XPS spectra of CCS before and after the adsorption of Li₂S₆, highlighting the strong interactions between CCS and lithium polysulfides. N 1s spectrum **(Figure S4(b))** shows a significant decrease in the peak intensity at 401.1 eV, which corresponds to C-NH₃⁺, after the adsorption of polysulfides. Concurrently, a new peak appears at 402.8 eV, assigned to Li–N bonds. This indicates a strong interaction between the C-NH₃⁺ groups and the lithium polysulfides. Additionally, C 1s **(Figure S4(a))** and O 1s **spectra (Figure S4(c))** reveal changes in the peak intensities of C-O and C=O groups. The trend of these changes aligns with the polysulfide adsorption strength, with the following order of adsorption affinity: C-NH₃⁺ > C-NH₂ > O=C-N, and OH > C=O > O-C-O. These functional groups exhibit a strong affinity for polysulfides, which likely leads to the coverage of these groups by excessive polysulfide adsorption, resulting in the weakened detection of these functional groups. These results demonstrate that the CCS binder interacts strongly with lithium polysulfides through electrostatic interactions, hydrogen bonding, and coordination between the lone pairs of nitrogen and oxygen atoms in CCS and Li⁺ in lithium polysulfides or Li₂S₆.^[55, 57, 58]^

Furthermore, to visually evaluate the interaction capacity of four binders with polysulfides, lithium-polysulfide adsorption experiments were conducted by immersing equivalent amounts of each binder material into a Li₂S₆ solution. As shown in **Figure 4e,** the binders shown different capabilities toward adsorption of polysulfides. The CMC/SBR result in lighter yellow color in comparison with the darker yellow color of control (1 mM Li_2_S_6_) and that of the PVDF binder. Remarkably, the Li_2_S_6_ solution treated with CCS and CCS-MWCNTs is nearly colorless as almost all polysulfides are adsorbed by the binders, indicating the stronger polysulfide adsorption capability of CCS and CCS-MWCNTs compared to that of CMC/SBR and PVDF binders. This is owing to the factor that chitosan contains rich amino/acetamido groups and secondary hydroxyl groups, offering strong chemical adsorptions of polysulfides than the CMC/SBR binder. The UV-vis absorption spectra for the supernatants from the adsorption experiments are presented in **Figure 4f**. The absorbance at 420 nm corresponds to the polysulfide specie of S_6_²⁻.^[59]^ The much lower absorbance for CCS-based samples at 420 nm than those of PVDF and CMC/SBR binders evidently supports the extraordinary polysulfide adsorption capability.


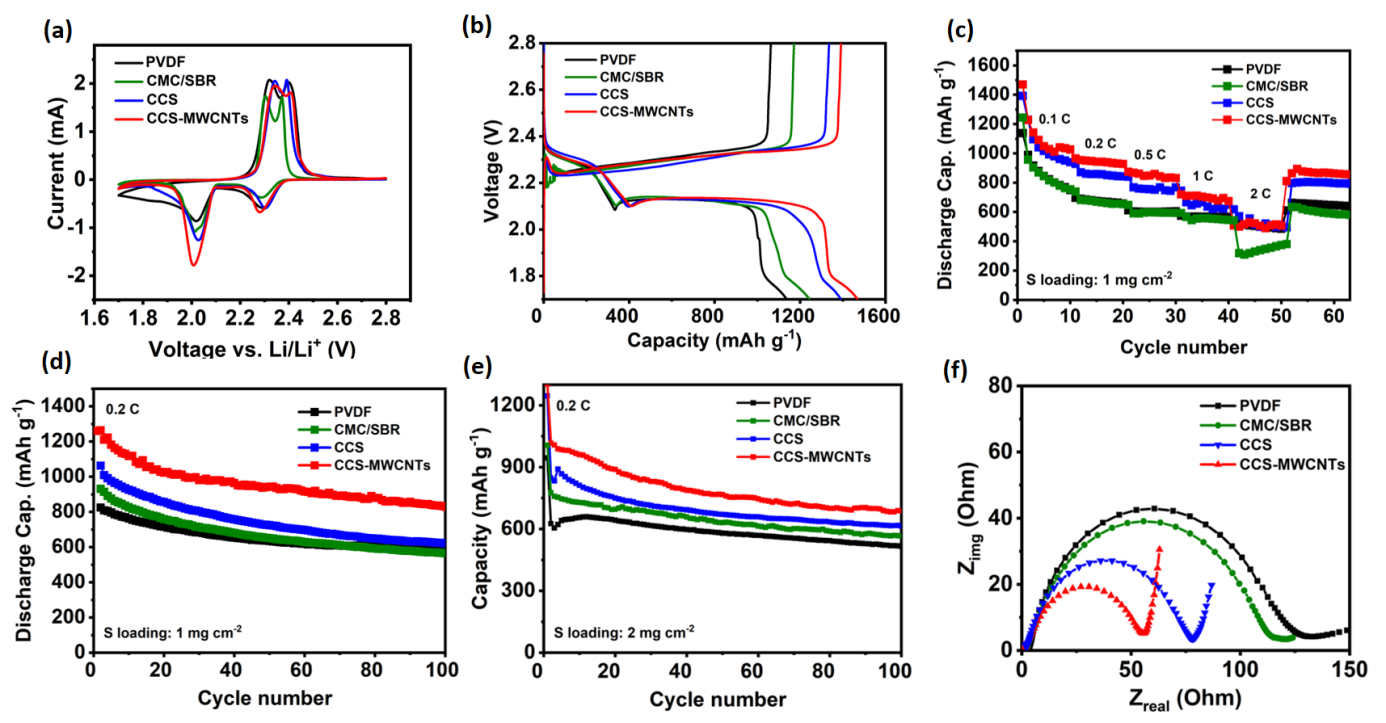
**Figure 5**. Electrochemical performances of Li-S batteries with different binders: The sulfur loading for (a-d, f) is 1 mg cm^-2^. (a) CV profiles, (b) voltage-capacity profiles for the first discharge and charge at 0.1 C, (c) rate capabilities, and (d) cycling performance at 0.2 C. (e) Cycling performance at 0.2 C of cathodes with sulfur loading of 2 mg cm^-2^. (f) Nyquist plots for Li-S batteries using different binders. (1 C=1675 mA g^-1^).

Cyclic voltammetry (CV) curves can intuitively reveal the electrochemical reactions occurring in the electrode and the reversibility of its charge-discharge processes. The CCS-MWCNT binder only performed the capacitive behavior and contribute to neglected capacity (**Figure S5**). **Figure 5a** shows the CV curves of four electrodes with the same sulfur loading (1 mg cm^-2^). For the reduction curves, the reduction peaks around 2.3 V correspond to the conversion of elemental sulfur to higher-order polysulfides, while the reduction peaks between 2.0-2.1 V correspond to the conversion of higher-order polysulfides to lower-order polysulfides.^[60]^ On the oxidation curves, the oxidation peaks between 2.3-2.5 V correspond to the conversion of lithium sulfide to elemental sulfur.^[61]^ Comparing the four curves, the CCS-MWCNTs-based electrode exhibits the highest reduction peak intensity, indicating the highest electrochemical reaction rate and the highest utilization of active material. Moreover, the reduction peak intensity of the CCS-MWCNTs-based electrode is comparable to its oxidation peak, whereas the reduction peaks of the other three electrodes are significantly lower than their oxidation peaks, indicating that the charge-discharge efficiency of the CCS-MWCNTs-based electrode is higher than the others. This suggests that the CCS-MWCNTs-based electrode has excellent cycling reversibility, demonstrating its potential for outstanding cycling performance.

The initial galvanostatic charge and discharge curves of the cathodes with four different binders are shown in **Figure 5b**. In accordance with the CV results, all sulfur cathodes demonstrate the typical two-plateau discharge profile of conventional Li−S batteries, i.e., the conversion of sulfur to highly soluble long-chain polysulfides at around 2.3 V and then the transformation to less-soluble or insoluble Li_2_S_2_/Li_2_S at around 2.1 V. At low current density (0.1 C), the polarization (i.e., the potential difference between reduction and oxidation) are similar for all cathodes with different binders, consistent with the CV profiles. Compared to that of the PVDF and CMC/SBR binders, Li−S batteries using chitosan binder exhibit obviously higher capacity. The CCS-MWCNTs-based cathode delivers the initial capacity of 1470 mAh g^-1^, corresponding to 88% of sulfur utilization. In addition, from the galvanotactic discharge processes it can be observed that the potential of the CCS-MWCNT cells is higher than that of the PVDF cells when the cells discharged to the same capacity (e.g. 2.136 V for CCS-MWCNTs-based cell vs 2.121 V for PVDF-based cell when discharged to capacity of 800 mAh/g). Meanwhile, in the charge processes, the potential of the CCS-MWCNT cells is lower than that of the PVDF cells when charged to the same capacity (e.g. 2.319 V for CCS-MWCNTs-based cell vs 2.334 V for PVDF-based cell when charged to capacity of 800 mAh/g). These results indicate that the CCS-MWCNT cells exhibited the lower polarization during both discharge and charge. Moreover, the strong chemical interaction between polysulfides and CCS is expected to enhance the Li_2_S precipitation by the functional groups of CCS. To verify it, Li_2_S deposition measurements on Ketjenblack electrodes with different binders, PVDF and CCS-MWCNTs were conducted to study the Li_2_S precipitation (**Figure S6**). The Li_2_S deposition capacity can be calculated based on the electrodepositing potentiostatical profiles from long-chain polysulfides Li_2_S_8_ to Li_2_S.^[62]^ The deposition capacity for Li_2_S precipitation was increased from 69 mAh g_s_^−1^ for PVDF binder to198 mAh g_s_^−1^ for the CCS-MWCNT binder, leading to187% of increment. Meanwhile the Li_2_S precipitation time at peak current density was increased from 175 seconds for PVDF binder-based electrodes to 580 seconds for CCS-MWCNT binder-based electrodes, contributing to more Li_2_S precipitation. Thus, the improved capacity of the CCS-MWCNTs-based cathodes observed from **Figure 5b** are assumed not only to its strong polysulfides adsorption capability and improved electrical conductivity by MWCNTs, but also to the enhanced Li_2_S precipitation by CCS-MWCNTs. It should be noted that the relatively low peak current density for CCS-MWCNTs indicate the increased Li_2_S precipitation is ascribed to the enhanced polysulfides adsorption and uniform Li_2_S precipitation rather than the kinetical conversion process.

**Figure 5c** presents the rate capability, an important parameter that reflects the battery operation under different current densities. The cathodes with commercial PVDF binder show rapid capacity decrease from 1138 mAh g^-1^ to 574 mAh g^-1^ when increasing the current densities from 0.1 C to 1 C. CMC/SBR-based cathodes perform similarly with PVDF-based cathodes. In contrast, chitosan-based cathodes exhibit much higher reversible capacities under various rates. In addition, the cathodes with CCS-MWCNTs present higher specific capacity than modified chitosan. Even at 1 C, the CCS-MWCNTs-based cathodes demonstrate 720 mAh g^-1^, which is 50% of the capacity at 0.1 C and much higher than that of the other binders. At 2 C, the similar capacities for CCS-MWCNT, CCS and PVDF binders are presumed to be the poor kinetics mainly limited by the sluggish conversion between short-chain polysulfide to lithium sulfide. The CCS-based binders have the ability to chemically suppress polysulfide shuttling, but is not expected to work as an electrocatalyst for the above sluggish conversion, which can be also supported by the relatively low peak current density of Li_2_S precipitation during potentiostatical measurements. Therefore, smaller difference or no obvious differences between CCS-MWCNTs and CCS when increasing the current density can happen. The lower capacity for CMC/SBR binder than other binders can be due to its poorer interfacial electrochemical resistance, which was also found in previous works.^[63]^ When the rate is returned to 0.2 C, a high specific capacity of over 800 mAh g^-1^ is still retained for the CCS-MWCNTs-based cathodes. The excellent rate capability for the CCS-MWCNT binders indicates the enhanced kinetics of discharge/charge by the MWCNTs and rich functional groups of chitosan.

High uniformity and structural integrity of cathodes are essential for long-term and stable battery operation. To evaluate the cathode stability using different binders, the long-term galvanostatic discharge-charge cyclic stability was further investigated by discharge-charge cycling tests at 0.2 C and 0.5 C for 100 cycles, respectively. As seen in **Figure 5d** and **Figure S7**, the capacity for CCS-MWCNTs-based cathodes after 100 cycles at 0.2 C remains at 830 mAh g^-1^, which is much higher than that of cathodes with PVDF, CMC/SBR and CCS binders. At 0.5 C, the initial discharge capacity of CCS-MWCNT cathodes reaches 1040 mAh g^-1^, corresponding to 60% of the theoretical capacity at such relatively high current density, and still persists at 826 mAh g^-1^ after 100 cycles. In comparison, cathodes with PVDF, CMC/SBR and modified chitosan binder exhibit lower capacity. The long-term cycling of PVDF and CCS-MWCNT cells (**Figure S8**) further demonstrates the improved capacity and cyclic stability over 400 cycles at 0.5 C. These improvements are not only attributed to the improved kinetics, strong polysulfides adsorption and facilitated Li_2_S precipitation by chitosan covalent bonded MWCNTs binder, but also can be ascribed to the crack-free homogeneity of CCS-MWCNTs-based cathodes, leading to more uniform electrochemical reactions at electrode-electrolyte interfaces than those using the PVDF and CMC/SBR binders.

Furthermore, increasing the areal sulfur loading is crucial for obtaining higher practical energy density. To demonstrate potential practical applications of chitosan binders, a higher sulfur loading of 2.0 mg cm^−2^ was investigated for comparing the cyclic stability of cathodes prepared using different binders. As shown in **Figure 5e**, the cathodes with CCS-MWCNTs binder exhibit initial capacity of 1300 mAh g^-1^, corresponding to 77% of the theoretical capacity. The reversible capacity remains at 690 mAh g^-1^ after 100 cycles at 0.2 C, exceeding the capacity and stability of the cathodes using commercial PVDF, CMC/SBR binders and modified chitosan binder. These results strongly demonstrate that the CCS-MWCNT binder is efficient in chemically trapping polysulfides and improving kinetics.

Electrochemical impedance spectroscopy (EIS) measurements were carried out to investigate the internal resistance and kinetics of the cathodes with different binders as shown in **Figure 5f**. Each curve shows the semicircle in the high-frequency region and the linear session in the low-frequency region, corresponding to the interfacial charge transfer resistance (R_c_) and mass-diffusion controlled Warburg resistance (R_w_), respectively. The interfacial charge transfer resistance for different binder-based cells follows the order of R_c_ (CCS-MWCNTs) < R_c_ (CCS) < R_c_ (CMC/SBR) < R_c_ (PVDF). The results indicate that the electrochemical charge transfer resistance of the battery based on the CCS binder is significantly lower than that of PVDF and CMC/SBR. This suggests that lithium ions exchange charges more easily at the electrochemical interface composed of the active material and the CCS binder. One reason for this is that electrodes prepared using the CCS binder are more compact and freer of obvious cracks, creating a continuous and intact electronic conduction network with lower electronic resistance. Additionally, the negatively charged functional groups on the CCS surface facilitate the adsorption of lithium ions, promoting their transport. The combined effects of these factors enhance charge exchange at the electrochemical interface, reducing the charge transfer resistance. Furthermore, the composite CCS binder with MWCNTs further reduces the electrode resistance, resulting in even lower electrochemical charge transfer resistance compared to modified CCS. The above electrochemical results align with the EIS results, further confirming the beneficial impact of a dense and uniform electrode structure and the incorporation of MWCNTs on enhancing the conversion efficiency of the active material and improving charge-discharge efficiency.


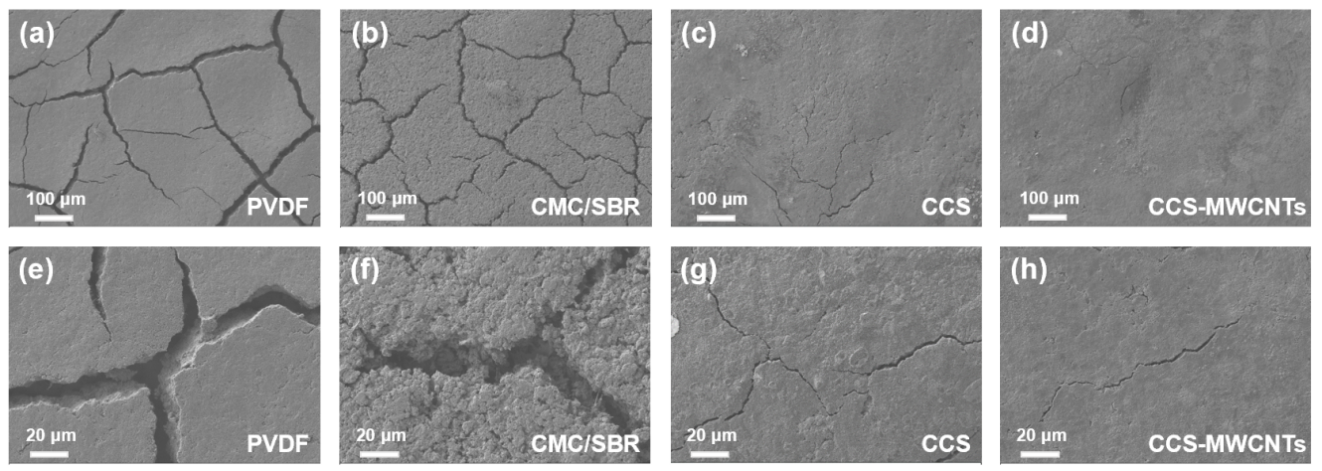


**Figure 6**. SEM images of the Li-S battery cathodes (sulfur loading 1 mg cm^-2^) prepared by various binders after cycling tests at 0.2 C.

Post-mortem analysis of cathode structures was then performed to validate the electrodes damage after cycling. **Figure 6** presents the SEM images of the galvanostatic discharge-charge tested electrodes from the four different binders (PVDF, CMC/SBR, CCS, and CCS-MWCNTs) after 100 cycles at a rate of 0.2 C. Notably, the CCS-MWCNTs-based electrode maintains optimal structural integrity post-cycling. In contrast, the PVDF-based electrode exhibits deepening cracks and an increase in fine fissures. The CMC/SBR-based electrode also shows a certain degree of crack propagation. Although the CCS-based electrode displays microcracks, as observed in **Figure 6g**, these cracks are shallower compared to those in the CMC/SBR electrode, with a uniform quality in the surrounding area, indicating that the CCS binder facilitates a more uniform dispersion of the electrode materials. **Figure 6h** reveals that the CCS-MWCNTs-based electrode contains only a single shallow crack and retains a dense surface, demonstrating good cycling stability. To quantitatively compare the integrity of the electrodes, defect areas were analyzed before and after cycling (**Figure S9**). The results indicate that the PVDF-based electrodes exhibit poor integrity, with defect rates reaching 4.43%, comparable to those of CMC/SBR (4.85%), and significantly higher than those of CCS (0.17%) and CCS-MWCNTs (0.04%). The superior integrity of the CCS-MWCNTs-based electrode can be attributed to the incorporation of MWCNTs. The covalent bonds formed between MWCNTs and CCS enhance the structural robustness. These findings further validate the advantages of the CCS-MWCNTs composite binder in improving electrode structural integrity, facilitating homogeneous electrochemical reactions, and enhancing the cyclic stability of Li-S batteries.

# 3. Conclusion

In conclusion, this work developed a novel composite binder for lithium-sulfur batteries by integrating catechol-conjugation modified chitosan with modified multi-walled carbon nanotubes. The CCS-MWCNTs binder offers enhanced mechanical properties through covalently bonded MWCNTs, strong polysulfide adsorption and facilitated Li_2_S precipitation due to its rich amino and hydroxyl groups, effectively addressing the challenges like electrode cracking, polysulfide shuttling, and structural degradation in LSBs. The enhanced chemical and mechanical performance of the CCS-MWCNTs composite binder not only contributes to its superior electrochemical stability and rate capability compared to the conventional binders, but also underscores its potential in advancing LSB technology. Furthermore, the bio-based nature of chitosan and its water-processing slurry formulation highlights the promising sustainability and low environmental impact for next-generation metal-sulfur battery applications, paving the way for more efficient and greener electrochemical energy storage solutions.

# 4. Experimental Section/Methods

*Materials:* Chitosan (CS, Mw~100,000 Da, deacetylation degree 85% ) was purchased from Macklin Co., Ltd. 3, 4-dihydroxhydrocinnamic acid (HCA) and 1-(3-dimethylaminopropyl)-3-ethylcarbodiimide hydrochloride (EDC) were purchased from Tokyo Chemical Industry Co., Ltd. Polyvinylidene fluoride (PVDF, battery grade) was purchased from MTI Corporation, carboxymethyl cellulose (CMC, Mw~250K Da) was purchased from sigma-Aldrich, styrene-butadiene rubber (SBR) was purchased from MTI Corporation, MWCNTs (below 10 nm diameter, 5-15 µm length) was purchased from TCI Chemicals.

*Synthesis of Catechol-conjugated Chitosan (CCS):* 2.500 g of chitosan was dissolved in 250 ml dilute acetic acid and the pH was adjusted to approximately 5.2. 1.783 g of 1-(3-dimethylaminopropyl)-3-ethylcarbodiimide hydrochloride (EDC) and approximately 1.695 g of 3,4-dihydroxyhydrocinnamic acid (HCA) were dissolved in a mixed solution of 125 mL deionized water and 125 mL anhydrous ethanol, the pH was then adjusted to around 4.8 using 0.1 mol L^-1^ nitric acid. The mixture was stirred thoroughly, and then added dropwise to the chitosan solution, which was allowed to proceed for 12 hours. After that, 5 L of 0.2 g L^-1^ LiNO_3_ solution was prepared as the dialysis medium, and the pH was adjusted to 4.6-4.8 using nitric acid. The modified chitosan solution was placed into a dialysis membrane tube and dialyzed against the LiNO_3_ solution for 3 days, during which the dialysis solution was replaced every 4-6 hours. Then, the chitosan was further cleaned by dialysis in diluted nitric acid solution with a pH of 4.6-4.8 for twice. Lastly, the CCS was obtained by freeze drying of the purified solution. CCS aqueous binder solution with a concentration of 10 mg mL^-1^ was prepared by dissolving 100 mg of catechol-conjugated chitosan (CCS) in 10 mL of water.

*Modification of Multi-Walled Carbon Nanotubes:* 100 mg of MWCNTs were refluxed with 50 mL 65% concentrated nitric acid at 110 °C for 24 hours. After cooling down to room temperature, the residue was thoroughly washed with deionized water until neutral pH (~7.0), filtered, and dispersed in 100 mL deionized water. The sample was then sonicated for 1 hour, frozen in liquid nitrogen, and dried by freeze drying.

*CCS-MWCNTs Binder:* The freeze-dried MWCNTs were dispersed in water and sonicated for 1-2 hours to form a 0.15 mg mL^-1^ MWCNTs uniform dispersion. 5 mL of the MWCNTs dispersion was stirred at 1000 rpm, and 2.425 mL of the 10 mg mL^-1^ CCS aqueous solution was added dropwise. After the addition, the mixture was sonicated for 1 hour，heated to 60 ℃ and stirred for 4 hours to obtain the CCS-MWCNTs binder.

*Preparation of Electrode Material:* 200 mg of Ketjen black carbon (KB, EC-600 JD) and 800 mg of sublimed sulfur were dissolved in a mixture of carbon disulfide and isopropanol (v/v 1:1) and stirred at 50 °C overnight. The KB-sulfur (KB/S) mixture was then transferred into a container in argon, sealed tightly, and subjected to heat treatment at 155 °C for 12 hours.

*Characterization Methods*: The Fourier transform infrared (FTIR) spectra were tested on TensorFlow II (Bruker Optics, Inc.). The ^1^H NMR spectrum of CCS polymer was obtained using AVANCE NEO (Bruker Corporation). Thermogravimetric (TG) and thermogravimetric analysis (TGA) of CS, CCS, and CCS-MWCNTs were conducted using TG 209 F1 (NETZSCH GmbH, Germany). X-ray photoelectron spectroscopy (XPS) analysis of the CCS samples before and after adsorption of polysulfides was carried out using Escalab 250Xi (Thermo Fisher Scientific Inc.). The structures and morphologies of the cathodes were measured by scanning electron microscopy (SEM, LEO 1530 field emission SEM). TEM images of MWCNTs before and after modification were obtained on a JEOL-2100 TEM (JEOL GmbH, Germany) with an operation voltage of 200 kV. The samples of MWCNTs before and after oxidation treatment were dispersed in water by ultrasonication, and then the dispersion was dropped onto Cu grids and dried for analysis.

*Lithium-polysulfide Adsorption Tests:* Lithium sulfide (Li_2_S) and sulfur with a molar ratio of 1:5 were added into the mixed solution of 1,3-dioxolane (DOL) and dimethoxyethane (DME) (v/v 1:1), and stirred at 80 °C for a few days in an argon glove box to obtain the Li_2_S_6_ solution. Different binder materials with 10 mg for each were added in 4 mL of 1 mM Li_2_S_6_ solution in the glove box, respectively. After standing for 4 h, the supernatants and host material particles were separated by centrifugation. Then, the collected supernatant was added into a quartz cuvette, sealed tightly in the glove box, and analyzed using a UV-vis spectrophotometer (Agilent 8453, Agilent Technologies, Inc.).

Li_2_S precipitation tests: First, the CCS-MWCNT electrodes containing 30 wt% of CCS-MWCNTs and 70% of Super P carbon were prepared by casting the water-based slurry onto Al foil. The preparation of the PVDF electrodes was the same except using PVDF as binder and NMP as the solvent. Then, the CR2025 coin cells were assembled using the above electrodes, Li metal anodes, PP Celgrad separators. In each cell, 20 μL catholyte consisting of 0.2 M Li_2_S_6_ and 1 M LiTFSI in the mixture solvent of DOL and DME (1:1, v/v) was added to the cathode side, meanwhile 20 μL of anolyte with 1 M LiTFSI in DOL and DME (1:1, v/v) was added in the anode side. After assembly, the cells were aged for 2 h and then discharged galvanostatically at a current density of 0.1 C to 2.16 V vs. Li/Li^+^, followed by being discharged potentiostatically at 2.05 V vs Li/Li^+^ for Li_2_S nucleation and growth. The potentiostat discharge was run over 65000 s.

*Mechanical Tests:* The mechanical tests were performed on a computer-controlled electronic universal testing machine (UTM2502, SANS Technology Co., Ltd.) with a strain rate of 5 mm min^-1^. The size of the tensile specimen was 15 mm * 60 mm.

*Electrochemical Testing:* The electrode material, super P and binder (7:2:1 in mass) were mixed in water by stirring and milling to form a homogeneous slurry. The as-prepared slurry was coated onto aluminum foil by a [doctor-blade casting](https://onlinelibrary.wiley.com/doi/abs/10.1002/aenm.202102259?casa_token=c59ydVukctoAAAAA:xuReaHhA4gN5rEtQgvxYMgKg1pR6csNbIxvLJE_XOCXI2W2k-cA-DB_iDyWB46vLL9DXu2cVzhrMSyY) process on a compact tape-casting coater (MSK-AFA-III, MTI Corporation). The wet coating was first dried at room temperature, and then dried in an oven at 60 °C for 12 hours. After drying, the coated aluminum foil was calendared, and then cut into discs (12 mm in diameter) as the cathodes. The S mass loading was controlled at ~ 1.0 and 2.0 mg cm^-2^, respectively. The 2032-type coin cells were assembled in an argon-filled glove box, using a lithium metal disc (15.6 mm in diameter) as the anode, a Celgard 2400 polypropylene separator, and the electrolyte consisting of 1 M lithium bis(trifluoromethane)sulfonamide (LiTFSI) dissolved in DOL and DME (v/v, 1:1) with 2 wt% of lithium nitrate as additives. 35 µL of electrolyte was added in each coin cell. Galvanostatic charge/discharge (GCD) measurements such as long-term cycling tests at 0.2 C, 0.5 C and rate performances were carried out using an NEWARE Model battery testing system within a cutoff voltage window of 1.7-2.8 V. Electrochemical impedance spectroscopy (EIS) was performed in the frequency range of 0.1 Hz – 10^5^ Hz at room temperature using a Gamry Potentiostat/Galvanostat (Interface 1000) with amplitude of 5 mV. Cyclic voltammetry (CV) was performed using Gamry Potentiostat/Galvanostat at a scan rate of 0.1 mV s^-1^ within 1.7-2.8 V. All the measurements were conducted three times for reproducibility.

**Supporting Information**

Supporting Information is available from the Wiley Online Library or from the author.

Acknowledgements

Q. Gou and L. Lu contributed equally to this work. Q. Gou acknowledges the International Summer Student Programme (ISSP) of Helmholtz-Zentrum Berlin (HZB). The authors thank Ping Feng and Sijia Cao for the TEM analysis of MWCNTs. Q. Gou and L. Zhu acknowledge the funding support from the Fundamental Research Funds for the Central Universities (2232024Y-01) and the National Key Research and Development Program of China (2021YFB3703900). S. Lin thanks to the International Postdoctoral Exchange Fellowship Program between Helmholtz-Zentrum Berlin für Materialien und Energie, OCPC and Shanghai Jiao Tong University, School of Materials Science and Engineering.

Received: ((will be filled in by the editorial staff))
Revised: ((will be filled in by the editorial staff))
Published online: ((will be filled in by the editorial staff))

References

[1] P. G. Bruce, S. A. Freunberger, L. J. Hardwick, J. M. Tarascon, *Nat. Mater.* **2011**, *11*, 19-29.

[2] Y. M. Kang, A. Cao, R. Clément, S. L. Chou, S. Y. Lee, Z. Miao, *Battery Energy* **2022**, *1*, 20220200.

[3] G. G. Njema, R. B. O. Ouma, J. K. Kibet, P. V, *J. Renew. Energy* **2024**, *2024*, 1-35.

[4] N. Boaretto, I. Garbayo, S. Valiyaveettil-SobhanRaj, A. Quintela, C. Li, M. Casas-Cabanas, F. Aguesse, *J. Power Sources* **2021**, *502*, 229919.

[5] H. Wu, X. Gao, X. Chen, W. Li, J. Li, L. Zhang, Y. Zhao, M. Jiang, R. Sun, X. Sun, *Carbon Energy* **2023**, *6*, e422.

[6] M. Armand, P. Axmann, D. Bresser, M. Copley, K. Edström, C. Ekberg, D. Guyomard, B. Lestriez, P. Novák, M. Petranikova, W. Porcher, S. Trabesinger, M. Wohlfahrt-Mehrens, H. Zhang, *J. Power Sources* **2020**, *479*, 228708.

[7] S. Tiwari, V. Yadav, A. K. Poonia, D. Pal, *J. Energy Storage* **2024**, *94*, 112347.

[8] L. K. J. Ting, Y. Gao, H. Wang, T. Wang, J. Sun, J. Wang, *ACS Omega* **2022**, *7*, 40682-40700.

[9] R. Sun, J. Hu, X. Shi, J. Wang, X. Zheng, Y. Zhang, B. Han, K. Xia, Q. Gao, C. Zhou, L. Mai, *Adv. Funct. Mater.* **2021**, *31*, 2104858.

[10] F. Zou, A. Manthiram, *Adv. Energy Mater.* **2020**, *10*, 2002508.

[11] S. L. Chou, Y. Pan, J. Z. Wang, H. K. Liu, S. X. Dou, *Phys. Chem. Chem. Phys.* **2014**, *16*, 20347-20359.

[12] T. Nakazawa, A. Ikoma, R. Kido, K. Ueno, K. Dokko, M. Watanabe, *J. Power Sources* **2016**, *307*, 746-752.

[13] H. Su, C. Fu, Y. Zhao, D. Long, L. Ling, B. M. Wong, J. Lu, J. Guo, *ACS Energy Lett.* **2017**, *2*, 2591-2597.

[14] Y. Wu, M. Yang, Y. Zou, S. Hou, B. Hu, S. Wang, Y. Tao, C. Yang, *New J. Chem.* **2022**, *46*, 20129-20137.

[15] Y. Yang, J. Qiu, L. Cai, C. Liu, S. Wu, X. Wei, D. Luo, B. Zhang, X. Yang, K. N. Hui, J. Liu, Z. Lin, *ACS Appl. Mater. Interfaces* **2021**, *13*, 33066-33074.

[16] S. C. Mun, Y. H. Jeon, J. H. Won, *Prog. Nat. Sci.: Mater. Int.* **2024**, *34*, 194-206.

[17] N. Lingappan, L. Kong, M. Pecht, *Renew. Sustainable Energy Rev.* **2021**, *147*, 111227.

[18] D. Bresser, D. Buchholz, A. Moretti, A. Varzi, S. Passerini, *Energy Environ. Mater.* **2018**, *11*, 3096-3127.

[19] W. Wang, X. Yue, J. Meng, X. Wang, Y. Zhou, Q. Wang, Z. Fu, *J. Phys. Chem. C* **2018**, *123*, 250-257.

[20] G. T. Pace, H. Wang, J. F. Whitacre, W. Wu, *Nano Select* **2021**, *2*, 939-947.

[21] L. Chen, Z. Jiang, X. Luo, Y. Zhong, H. Wang, X. Wang, Q. Huang, X. Liu, S. Kawaguchi, W. Li, *Chem. Eng. J.* **2021**, *421*, 127769.

[22] W. Bao, Z. Zhang, Y. Gan, X. Wang, J. Lia, *J. Energy Chem.* **2013**, *22*, 790-794.

[23] H.-t. Xu, Z.-y. Zou, B. Tang, Z. Chen, H.-r. Zhang, J.-c. Chen, G.-l. Cui, *Acta Polymerica Sin.* **2021**, *52（2）*, 166-175.

[24] Y.-Q. Lu, J.-T. Li, X.-X. Peng, T. Zhang, Y.-P. Deng, Z.-Y. Wu, L. Deng, L. Huang, X.-D. Zhou, S.-G. Sun, *Electrochem. Commun.* **2016**, *72*, 79-82.

[25] S. Chen, H. Y. Ling, H. Chen, S. Zhang, A. Du, C. Yan, *J. Power Sources* **2020**, *450*, 227671.

[26] Z. W. Seh, Y. Sun, Q. Zhang, Y. Cui, *Chem Soc Rev* **2016**, *45*, 5605-5634.

[27] S. Liang, J. Zhang, C. Jia, Z. Luo, L. Zhang, *Carbon* **2024**, *222*, 118807.

[28] N. Patra, P. Ramesh, V. Donthu, A. Ahmad, *J. Mater. Sci.: Mater. Electron.* **2024**, *19*, 19.

[29] W. Wang, C. Xue, X. Mao, *Int J Biol Macromol* **2020**, *164*, 4532-4546.

[30] H. Chen, M. Ling, L. Hencz, H. Y. Ling, G. Li, Z. Lin, G. Liu, S. Zhang, *Chem. Rev.* **2018**, *118*, 8936-8982.

[31] D. Lingait, R. Rahagude, S. S. Gaharwar, R. S. Das, M. G. Verma, N. Srivastava, A. Kumar, S. Mandavgane, *Int. J. Biol. Macromol.* **2024**, *257*, 128676.

[32] Y. Chu, N. Chen, X. Cui, A. Liu, L. Zhen, Q. Pan, *J. Energy Chem.* **2020**, *46*, 99-104.

[33] J. Feng, H. Yi, Z. Lei, J. Wang, H. Zeng, Y. Deng, C. Wang, *J. Energy Chem.* **2021**, *56*, 171-178.

[34] A. Mayrén, J. J. Alcaraz-Espinoza, A. Hernández-Sánchez, I. González, G. Ramos-Sánchez, *Electrochim. Acta* **2024**, *480*, 143917.

[35] Y. Chen, N. Liu, H. Shao, W. Wang, M. Gao, C. Li, H. Zhang, A. Wang, Y. Huang, *J. Mater. Chem. A* **2015**, *3*, 15235-15240.

[36] Y. Yuan, Z. Li, H. Lu, H. Cheng, D. Zheng, Z. Fang, *New J. Chem.* **2020**, *44*, 10648-10653.

[37] S. Kim, M. Cho, Y. Lee, *Adv. Funct. Mater.* **2020**, *30*, 112347.

[38] R. Deng, M. Wang, H. Yu, S. Luo, J. Li, F. Chu, B. Liu, F. Wu, *Energy Environ. Mater.* **2022**, *5*, 777-799.

[39] C. Yang, Y. Tang, Y. Zhang, X. Zeng, M. Dai, L. Liu, *New J. Chem.* **2024**, *48*, 8569-8573.

[40] L. Yu, L. Xu, C. Shearer, X. Zhou, L. Lu, *Adv. Sustain. Syst.* **2023**, *7*, 2300061.

[41] P. Ganji, S. Nazari, A. A. Zinatizadeh, S. Zinadini, *J. Water Process Eng.* **2022**, *48*, 102922.

[42] L. A. Alshabanah, S. M. Gomha, L. A. Al-Mutabagani, T. Z. Abolibda, N. A. A. El-Ghany, W. El-Enany, A. K. El-Ziaty, R. S. Ali, N. A. Mohamed, *Polymers (Basel)* **2021**, *13*, 1728.

[43] F. F. Hassan, F. F. Karam, *IOP Conf. Ser.: Earth Environ. Sci.* **2021**, *790*, 012008.

[44] G. Ke, W. C. Guan, C. Y. Tang, Z. Hu, W. J. Guan, D. L. Zeng, F. Deng, *Chin. Chem. Lett.* **2007**, *18*, 361-364.

[45] G. Ke, W. Guan, C. Tang, W. Guan, D. Zeng, F. Deng, *Biomacromolecules* **2007**, *8*, 322-326.

[46] P. Abrica-Gonzalez, J. A. Zamora-Justo, A. Sotelo-Lopez, G. R. Vazquez-Martinez, J. A. Balderas-Lopez, A. Munoz-Diosdado, M. Ibanez-Hernandez, *Nanoscale Res Lett* **2019**, *14*, 258.

[47] J. Na, and Kunthavai Nachiyar Rb, *Int. J. Curr. Sci.* **2014**, *11*, 18-25.

[48] A. R. Narkar, E. Cannon, H. Yildirim-Alicea, K. Ahn, *Langmuir* **2019**, *35*, 16013-16023.

[49] C.-P. Chang, C. Wang, K.-J. Hung, Y.-S. Perng, *Taiwan J. Forest Sci.* **2010**, *25(3)*, 251-264.

[50] G. P. López, D. G. Castner, B. D. Ratner, *Surf. Interface Anal.* **2004**, *17*, 267-272.

[51] X.-Y. Rao, S.-F. Xiang, J. Zhou, Z. Zhang, X.-Y. Xu, Y.-Y. Xu, X.-C. Zhou, Z.-D. Pan, S.-C. Tan, S.-X. Dong, Z.-L. Wang, Y.-T. Wu, Y.-L. Zhou, X. Liu, Y. Zhang, S. Jiang, *Rare Metals* **2024**, *43*, 4132-4161.

[52] J. Wang, H. Wang, S. Jia, Q. Zhao, Q. Zheng, Y. Ma, T. Ma, X. Li, *J. Energy Storage* **2023**, *72*, 108372.

[53] Y. Bourlier, M. Bouttemy, O. Patard, P. Gamarra, S. Piotrowicz, J. Vigneron, R. Aubry, S. Delage, A. Etcheberry, *ECS J. Solid State Sci. Technol.* **2018**, *7*, P329-P338.

[54] Z. Wang, Y. Wang, W. Wang, X. Yu, W. Lv, B. Xiang, Y. B. He, *Front Chem* **2018**, *6*, 97.

[55] L.-C. Yin, J. Liang, G.-M. Zhou, F. Li, R. Saito, H.-M. Cheng, *Nano Energy* **2016**, *25*, 203-210.

[56] C. Zhang, H. Zhang, R. Li, Y. Xing, *RSC Adv.* **2017**, *7*, 48189-48198.

[57] W. Zhou, X. Xiao, M. Cai, L. Yang, *Nano Lett.* **2014**, *14*, 5250-5256.

[58] Z. W. Seh, Y. Sun, Q. Zhang, Y. Cui, *Chem. Soc. Rev.* **2016**, *45*, 5605-5634.

[59] L. Hencz, H. Chen, Z. Wu, X. Gu, M. Li, Y. Tian, S. Chen, C. Yan, A. S. R. Bati, J. G. Shapter, M. Kiefel, D.-S. Li, S. Zhang, *Green Energy Environ.* **2022**, *7*, 1206-1216.

[60] M. Agostini, D.-J. Lee, B. Scrosati, Y. K. Sun, J. Hassoun, *J. Power Sources* **2014**, *265*, 14-19.

[61] S. Lang, S. H. Yu, X. Feng, M. R. Krumov, H. D. Abruna, *Nat. Commun.* **2022**, *13*, 4811.

[62] D. Xie, Y. Xu, E. Härk, Z. Kochovski, X. Pan, X. Zhang, J. Schmidt, Y. Lu, *Mater. Today Energy* **2023**, *36*, 101344.

[63] J.-P. Yen, C.-C. Chang, Y.-R. Lin, S.-T. Shen, J.-L. Hong, *J. Electrochem. Soc.* **2013**, *160*, A1811-A1818.

A multifunctional aqueous composite binder is developed for Li-S batteries by using catechol-conjugated chitosan covalent bonded with multi-wall carbon nanotubes. The strong polysulfides adsorption and improved mechanical properties lead to crack-free electrodes and improved adhesion. The electrochemical performances with the composite binder demonstrate a high reversible capacity, superior cyclic stability and rate capability, significantly outperforming other commercial binders (e.g. PVDF).

Q. Gou, L. Lu, S. Lin, W. Zhang, Y.R. Ayllon, Z. Zhou, L. Zhu,* Y. Lu*

**Multifunctional Chitosan–Covalent Bonded Multi-walled Carbon Nanotubes Composite Binder for Enhanced Electrochemical Performances of Lithium-sulfur Batteries**


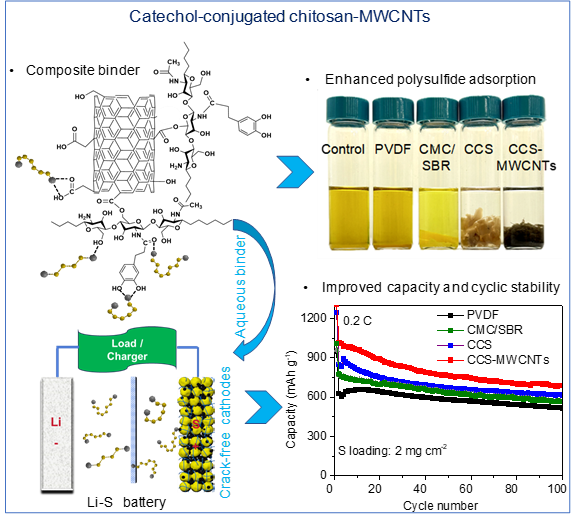


Supporting Information

**Multifunctional Chitosan–Covalent Bonded Multi-walled Carbon Nanotubes Composite Binder for Enhanced Electrochemical Performances of Lithium-sulfur Batteries**

*Qiuying Gou, Liqiang Lu, Shengxuan Lin, Wei Zhang, Yael Rodriguez Ayllon, Zhe Zhou, Liping Zhu,* Yan Lu,**


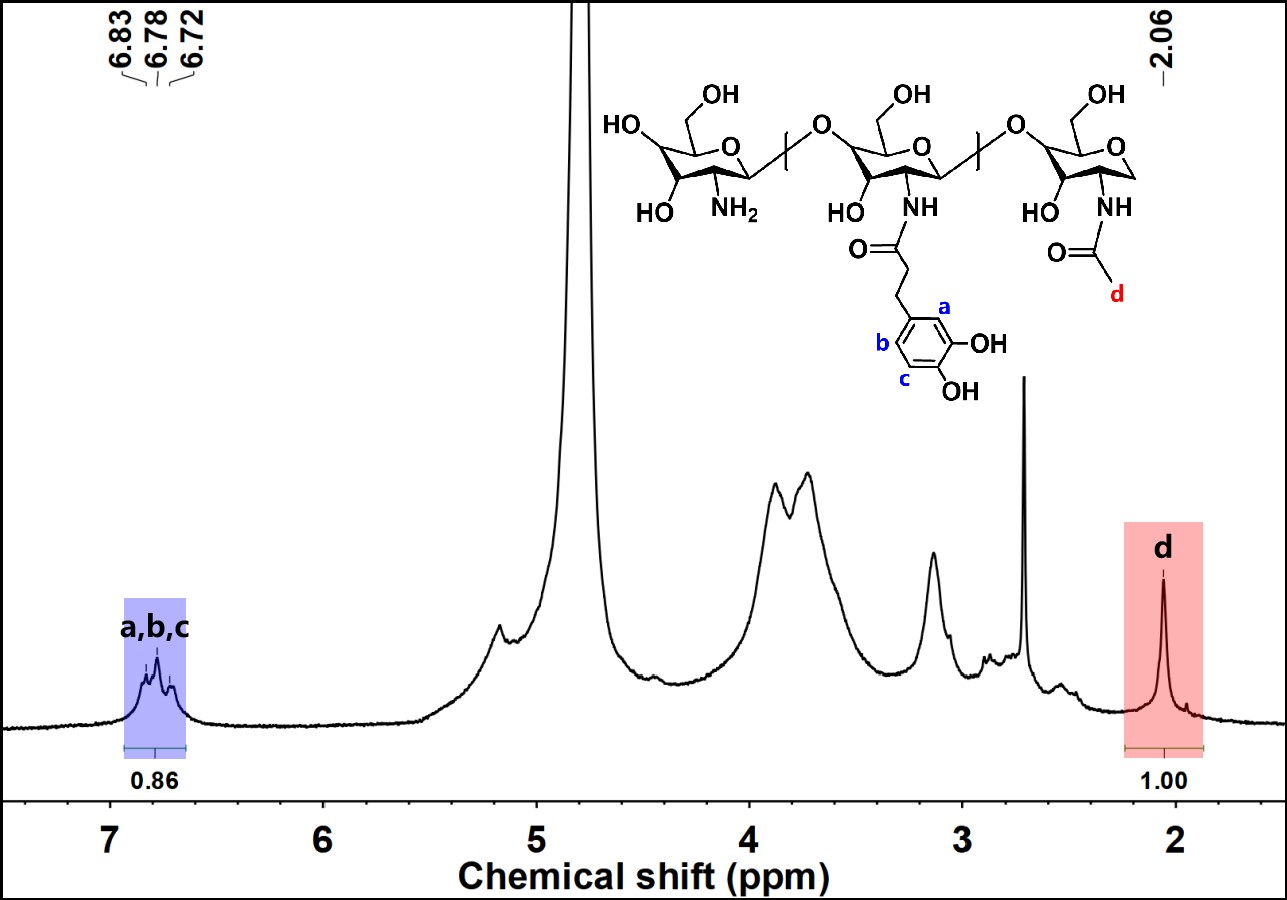


**Figure S1** NMR of the CCS binder.


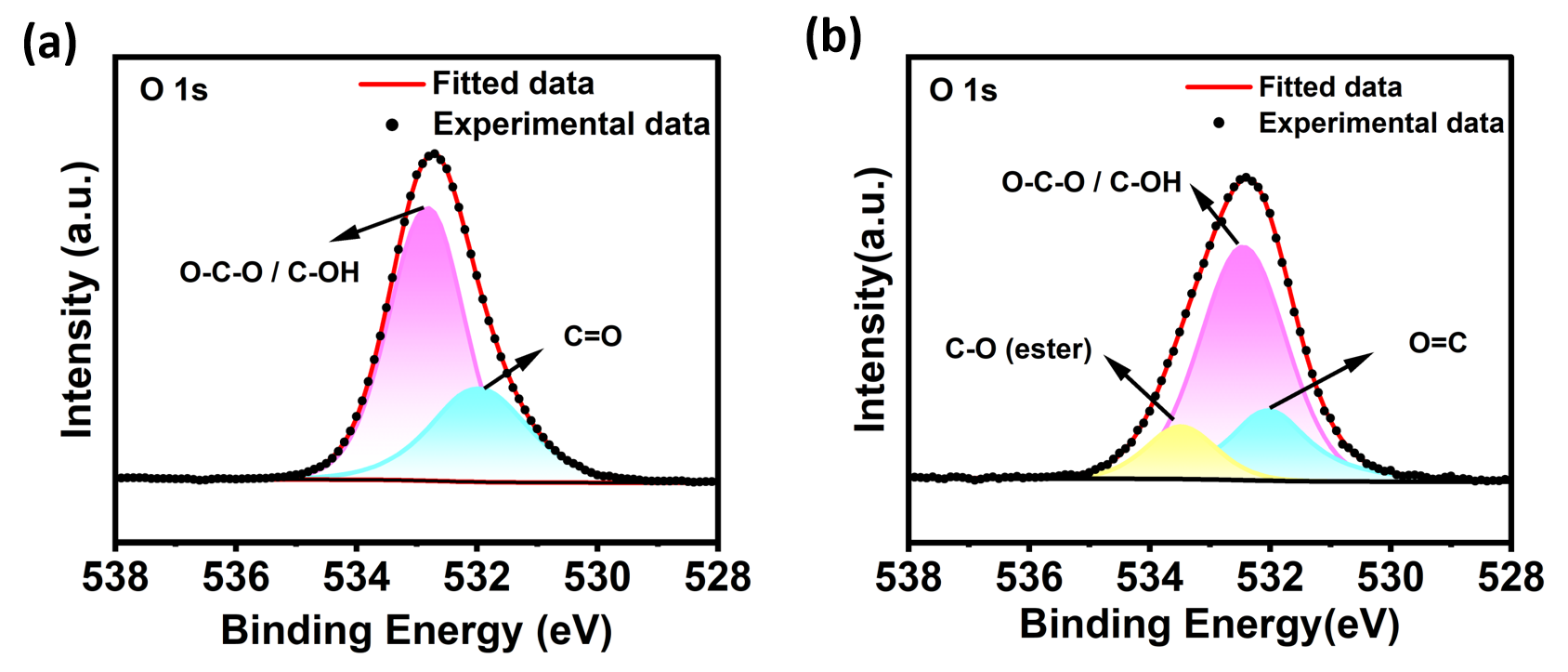


**Figure S2** XPS O 1s spectra of (a) CCS and (b) CCS-MWCNTs.


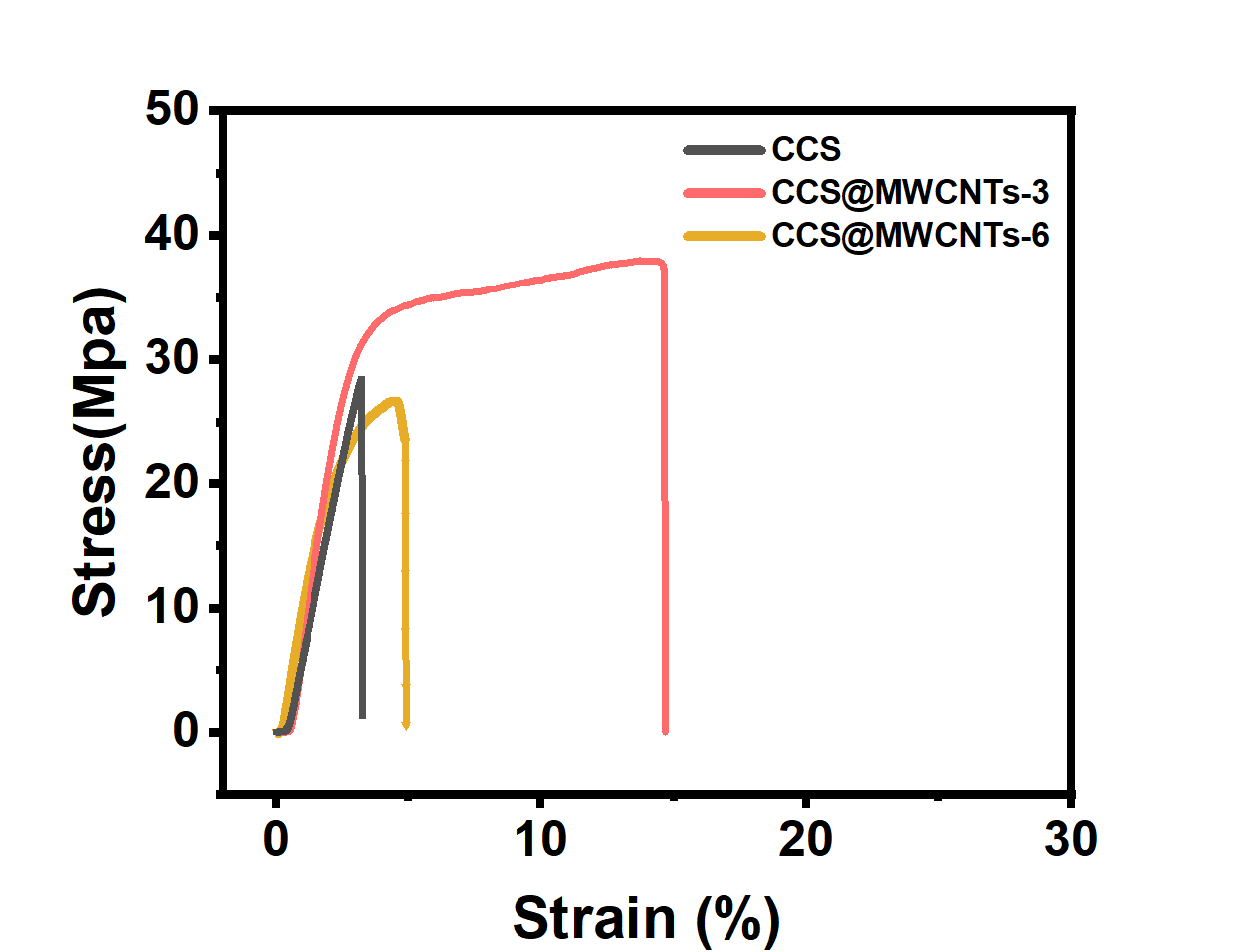


**Figure S3** Stress–strain curves of CCS binder films with two different MWCNT contents: 3 wt% (CCS@MWCNTs-3) and 6 wt% (CCS@MWCNTs-6)


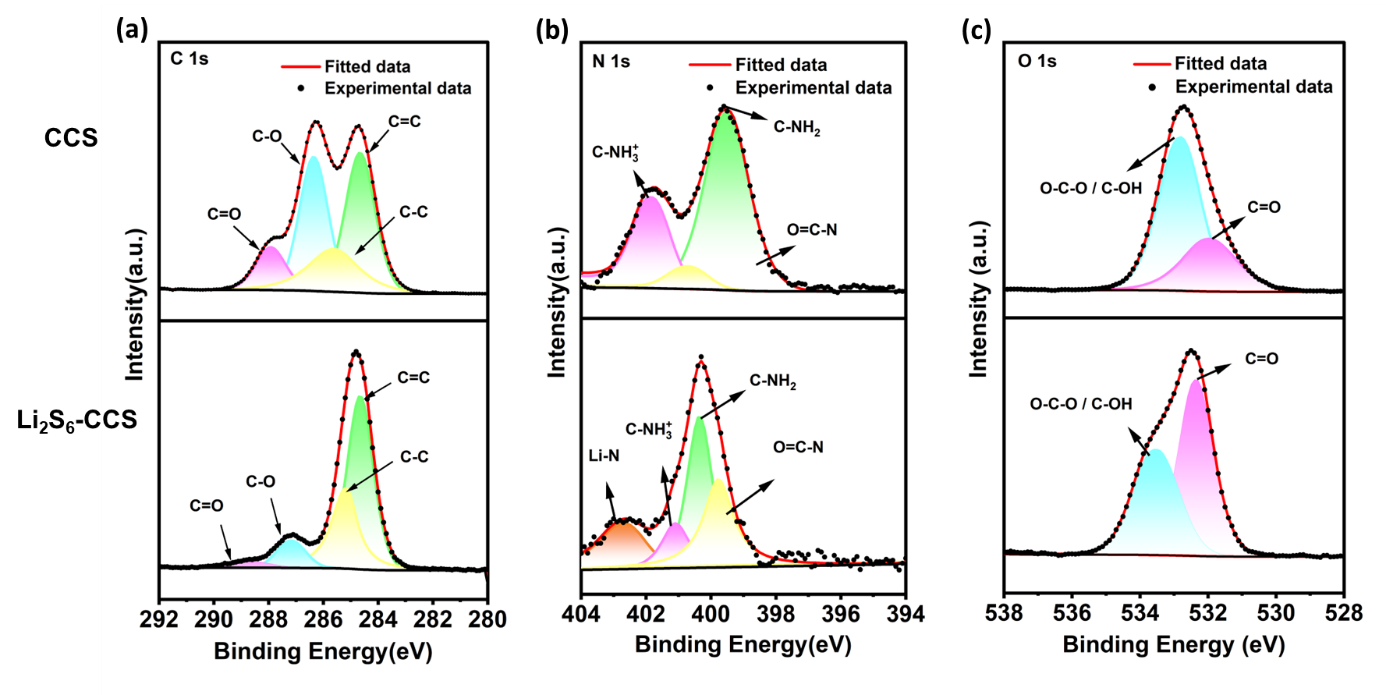


**Figure S4** XPS spectra of CCS and Li_2_S_6_-CCS: (a) C 1s, (b) N 1s, (c) O 1s.


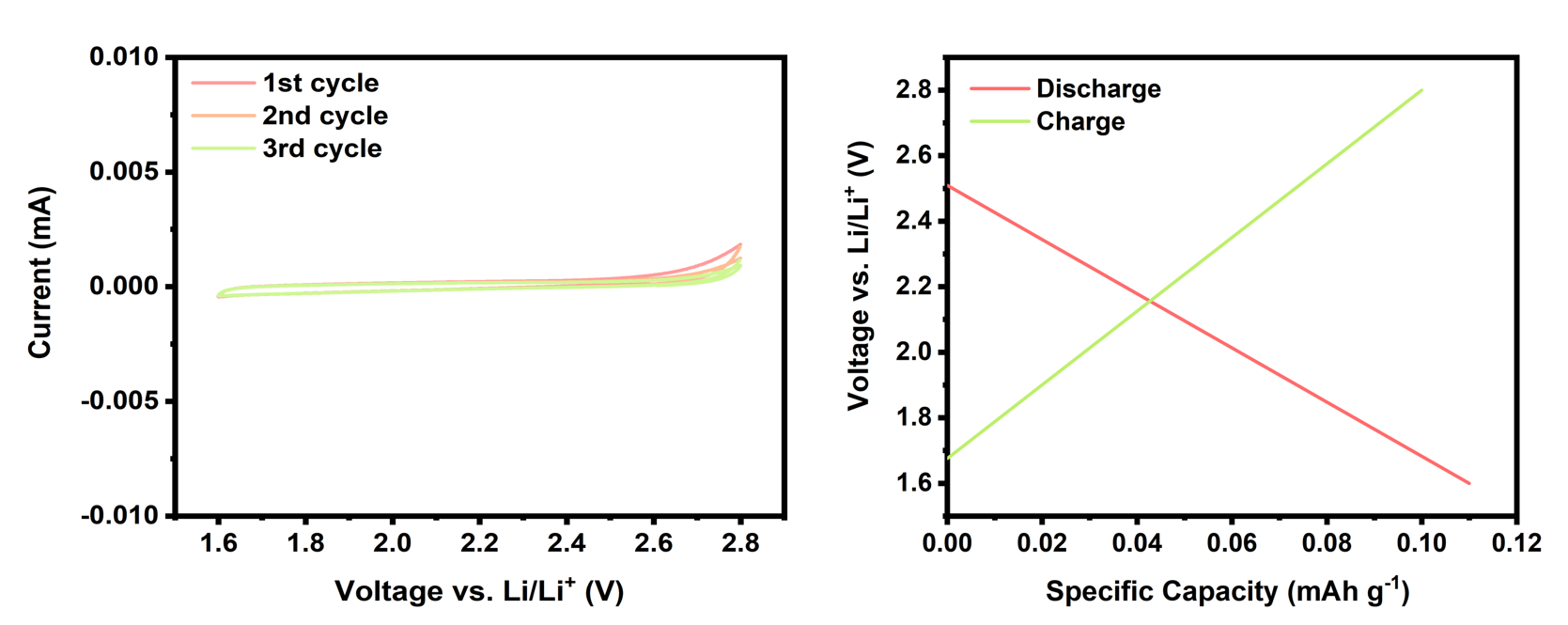


**Figure S5** CV curves and one-cycle galvanostatic charge/discharge curves of CCS-MWCNTs.


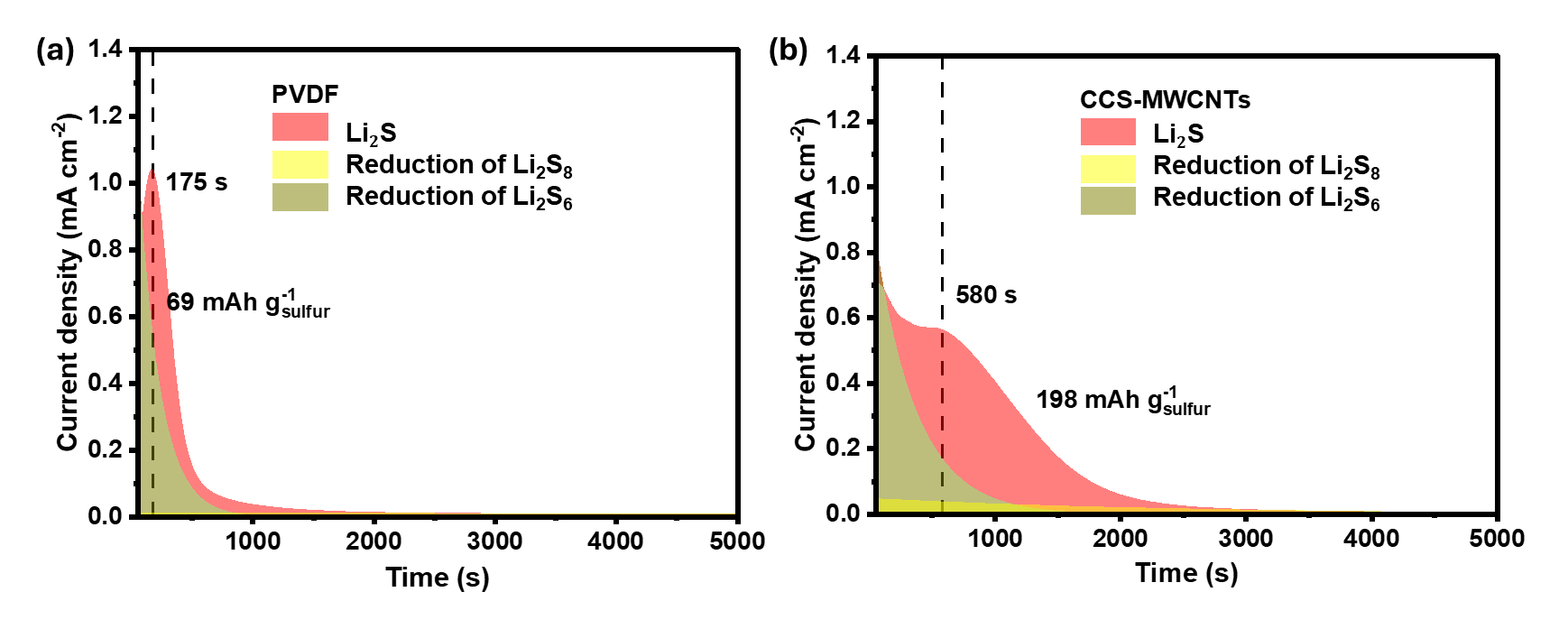


**Figure S6** Potentiostatic discharge profiles of Li_2_S precipitation on Ketjenblack electrodes with different binders (a) PVDF and (b) CCS-MWCNTs.


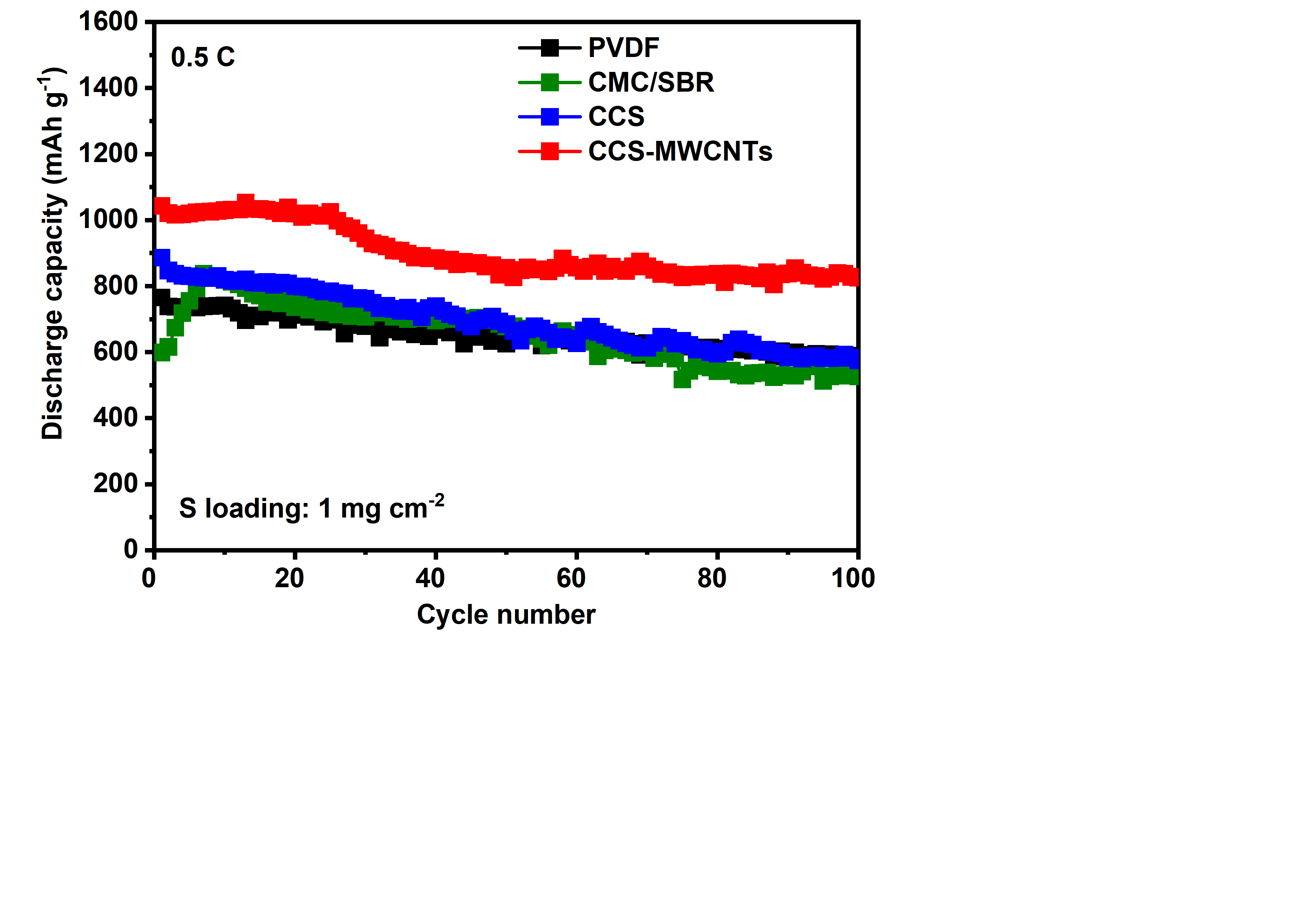


**Figure S7** The cyclic stability of cathodes with sulfur loading of 1 mg cm^-2^ prepared using different binders at a rate of 0.5 C.

**Figure S8** The long-term cycling of cathodes prepared using different binders at a rate of 0.5 C.


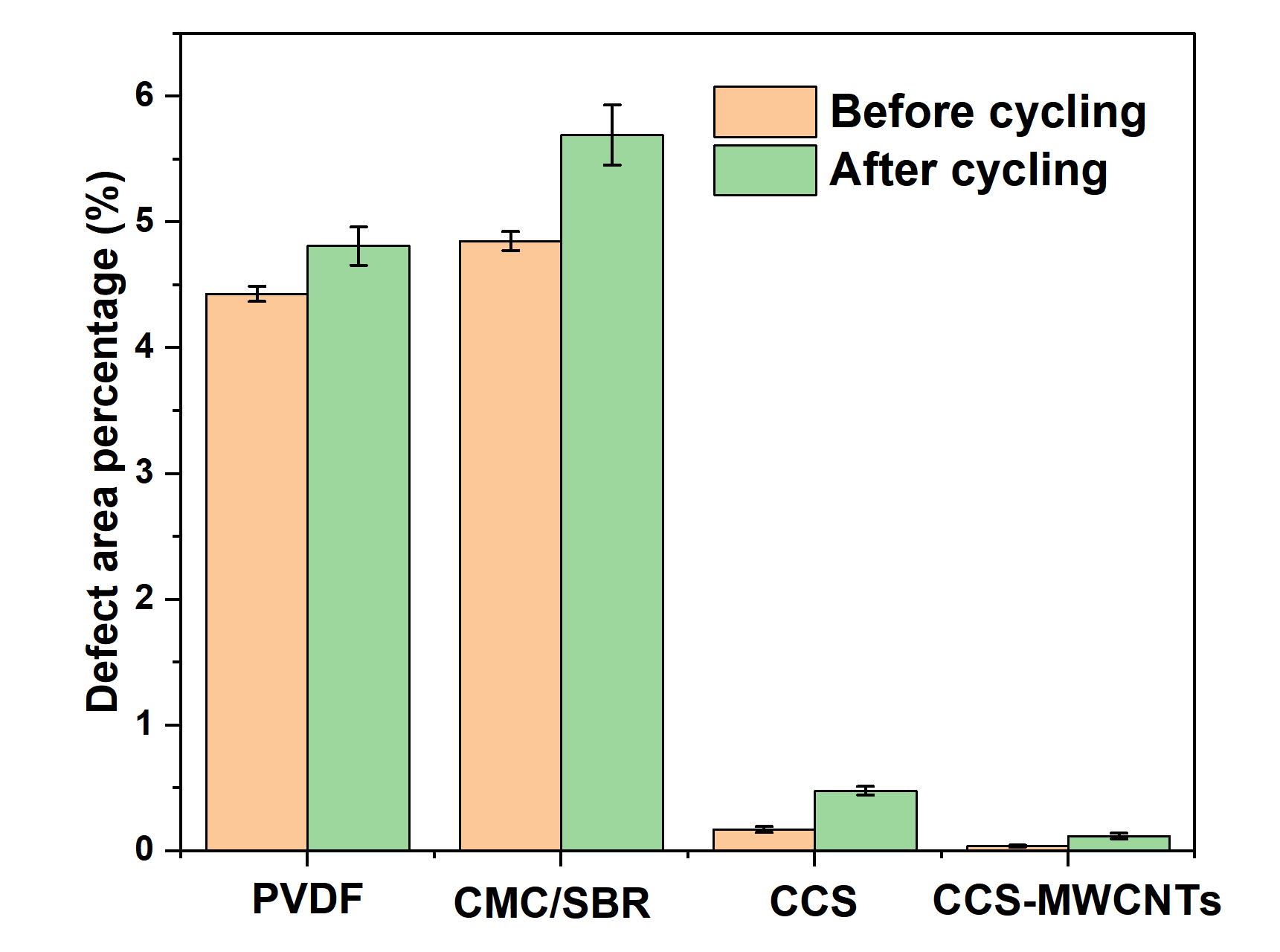


**Figure S9** Defect area percentage of different binders before and after cycling tests at 0.2 C.

**Table S1** Typical polymer binders and the resulted electrochemical performance of Li-S batteries.

| Binders | Cathodes | Capacity | Cyclic stability | 2C | Ref. |
| --- | --- | --- | --- | --- | --- |
| PEO | KBS | 800 mAh g^−1^  at 0.08 C | 0.08 C, 30 cycles,  598 mAh g^−1^ | / | [12] |
| PDAT | S-GO@C | 900 mAh g^–1^  at 0.1C | 0.1 C, 50 cycles,  837 mAh g^−1^ | / | [13] |
| 3M_90_ | Super P/S | 1239 mAh g^-1^  at 0.1 C | 0.1 C, 100 cycles,  772 mAh g^−1^  0.5 C, 500 cycles,  565 mAh g^−1^ | / | [14] |
| Pectin binder | ABS | \ | 0.5 C, 250 cycles,  660 mAh g^−1^ | 450 mAh g^-1^ | [23] |
| HMM/PAA | KBS | \ | 1 C, 300 cycles,  475 mAh g^−1^ | 577 mAh g^-1^ | [27] |
| CNC | Super P/S | \ | 1 C, 500 cycles,  485 mAh g^−1^ | / | [32] |
| ChMSA25 | Carbon Super P/S | 780 mAh g^-1^  at 0.1 C | 1C, 500 cycles,  296 mAh g^−1^  0.5C, 100 cycles, | 212 mA h g^−1^ | [34] |
| Chitosan | ABS | 1150 mAh g^−1^  at 0.1 C | 0.5 C, 100 cycles,  680 mAh g^−1^ | 600 mAh g^-1^ | [35] |
| PAM/PAA | Carbon Composite/S | 1008 mAh g^-1^  at 0.2 C | 0.2 C, 200 cycles,  600 mAh g⁻¹ | 500 mAh g^-1^ | [36] |
| CCS-MWCNTs | KBS (S~80wt%) | 1470 mAh g^-1^  at 0.1C | 0.2 C, 100 cycles,  830 mAh g^−1^  0.5 C, 400 cycles,  700 mAh g^−1^ | 515 mAh g^-1^ | This work |

Notes: PEO-polyethylene oxide, PDAT- poly(diallyldimethylammonium triflate), 3M-1,1-di-tert-butylperoxy-3,3,5-trimethylcyclohexane, HMM/PAA- hexamethylolmelamine/polyacrylic acid, CNC- chitosan-nitrocatechol, PAM/PAA- polyacrylamide/polyacrylic acid, GO-graphene oxide, ABS-acetylene black carbon-sulfur composite, ChMSA25- Chitosan – Methanesulfonic Acid (with 25% MSA addition).
